# Perception of Recorded Music With Hearing Aids: Compression Differentially Affects Musical Scene Analysis and Musical Sound Quality

**DOI:** 10.1177/23312165251368669

**Published:** 2025-08-25

**Authors:** Robin Hake, Michel Bürgel, Christophe Lesimple, Matthias Vormann, Kirsten C. Wagener, Volker Kuehnel, Kai Siedenburg

**Affiliations:** 1 Department of Medical Physics and Acoustics, 597451 Carl von Ossietzky Universität Oldenburg, Oldenburg, Germany; 2 87724 Sonova AG, Stäfa, Switzerland; 3 Hörzentrum Oldenburg gGmbH, Oldenburg, Germany; 4 Signal Processing and Speech Communication Laboratory, 27253 Graz University of Technology, Graz, Austria

**Keywords:** auditory scene analysis, Musical Scene Analysis, music, speech, compression, hearing aids, sound quality

## Abstract

Hearing aids have traditionally been designed to facilitate speech perception. With regards to music perception, previous work indicates that hearing aid users frequently complain about music sound quality. Yet, the effects of hearing aid amplification on musical perception abilities are largely unknown. This study aimed to investigate the effects of hearing aid amplification and dynamic range compression (DRC) settings on musical scene analysis (MSA) abilities and sound quality ratings (SQR) using polyphonic music recordings. Additionally, speech reception thresholds in noise (SRT) were measured. Thirty-three hearing aid users with moderate to severe hearing loss participated in three conditions: unaided, and aided with either slow or fast DRC settings. Overall, MSA abilities, SQR and SRT significantly improved with the use of hearing aids compared to the unaided condition. Yet, differences were observed regarding the choice of compression settings. Fast DRC led to better MSA performance, reflecting enhanced selective listening in musical mixtures, while slow DRC elicited more favorable SQR. Despite these improvements, variability in amplification benefit across DRC settings and tasks remained considerable, with some individuals showing limited or no improvement. These findings highlight a trade-off between scene transparency (indexed by MSA) and perceived sound quality, with individual differences emerging as a key factor in shaping amplification outcomes. Our results underscore the potential benefits of hearing aids for music perception and indicate the need for personalized fitting strategies tailored to task-specific demands.

## Introduction

Hearing aids have traditionally been designed to improve the intelligibility and clarity of speech signals (e.g., Popelka et al., 2016). This emphasis is rooted in the critical importance of speech communication in daily life, which has driven technological advancements and design priorities in hearing aid development. However, music perception presents a set of challenges and requirements that are not always adequately addressed by hearing aids optimized for speech. This has led to frequent complaints from hearing aid users about poor sound quality for music, often reporting that amplified music sounds unnatural or distorted (e.g., Greasley et al. 2020; Vaisberg et al., 2019). These complaints suggest that current hearing aid technologies may not fully accommodate the different set of acoustic demands, such as the wide frequency or dynamic range inherent to many classes of musical sounds (Chasin, 2012; Chasin & Russo, 2004; Lesimple et al., 2024). Such user experiences underscore the need for a more detailed understanding of how hearing aid processing parameters interact with the acoustic properties of music. Therefore, the present study aims to systematically investigate how different hearing aid settings impact perceived sound quality and behavioral measures of music perception. That is, by complementing sound quality ratings with selective listening tasks, this study seeks to provide a deeper understanding of the auditory experience of music among hearing aid users.

### Sound Quality With Hearing Aids

The literature suggests mixed results regarding hearing aid users’ assessment of musical sound quality. While some surveys have highlighted positive results—with 41% of respondents in one study reporting that hearing aids improve music enjoyment and only 6% indicating that music became less enjoyable (Leek et al., 2008), and another showing that hearing aids helped with both live and reproduced music (Madsen & Moore, 2014)—these findings are not universal. A survey conducted among professional and amateur musicians in German orchestras (Hake et al., 2024) found that 54% of regular hearing aid users rated the quality of their devices as “rather not sufficient” for the context of performing music, and 40% found them inadequate for music listening. Further, Looi et al. (2019) found that those with moderate to severe hearing loss tended to report less enjoyment and perceived music as less melodic when using their devices. Another survey conducted by Greasley et al. (2020) underscores these challenges, reporting that hearing aid users frequently experience feedback, distortion, and difficulties in live music settings—with 67% hearing aid users expressed to have difficulty listening to music. The cumulative impact of these issues has led many hearing-impaired musicians to prefer the sound quality and dynamics experienced without hearing aids, often removing their devices during musical performances (Vaisberg et al., 2019). This tendency of avoidance is further exacerbated by recommendations from some audiologists to forgo these devices for music listening altogether in cases of mild to moderate hearing loss (Greasley et al., 2020; Vaisberg, 2019). This range of experiences, from improved enjoyment to active avoidance, illustrates the need for a deeper understanding of the factors influencing music perception with hearing aids.

A critical component of modern hearing aids that may influence music perception is dynamic range compression (DRC). DRC provides level-dependent amplification tailored to the individual's hearing loss and compensates for the reduced dynamic range associated with sensorineural hearing loss—a phenomenon known as loudness recruitment (Steinberg & Gardner, 1937). The resulting adjustment of amplification enables soft sounds to become audible, while loud sounds are prevented from becoming uncomfortably loud. Slow DRC systems adjust the gain gradually, maintaining the natural temporal envelope of the input sounds. This helps preserve signal clarity and reduces harmonic and intermodulation distortion. Additionally, slow DRC preserves short-term level changes, aiding sound localization based on interaural level differences (Moore, 2008). However, these systems have drawbacks. For example, slow DRC may struggle with alternating voices of different levels and has a delayed gain adjustment (1–2 s), which may be problematic in situations with sudden large level changes. Therefore, it offers limited benefit in “listening in the dips” (perceiving “glimpses” of a target; Duquesnoy, 1983), because the gain does not increase significantly during brief dips in the input signal; a situation particularly problematic among hearing-impaired individuals, who already have a reduced ability to perceive sounds during brief dips in background noise (Duquesnoy, 1983; Peters et al., 1998, Siedenburg et al., 2020). It is reported that slow DRC are implemented in optional music modes for hearing devices (Moore, 2016).

Fast DRC systems, on the other hand, adjust the gain rapidly, thought to more closely approximate normal loudness perception and effectively compensate for frequency-dependent changes in loudness recruitment. Fast DRC can also better restore the audibility of weak sounds following intense sounds, facilitating the potential for listening in the dips. These systems perform well when two sources alternate with markedly different levels. In speech recognition, it has been shown that intelligibility at low speech levels is improved with fast DRC compared to slow DRC (Kowalewski et al., 2018). However, fast DRC can alter the natural shape of the temporal envelope of sounds and may distort sounds gliding in frequency if the DRC channels are formed using sharp, non-overlapping filters (Moore, 2008). Additionally, it can make background noises sound noisier, which can be annoying for new hearing aid users, and may introduce cross-modulation between voices in a mixture, reducing the ability to perceptually segregate streams (Madsen et al., 2015; Stone & Moore, 2004, 2007, 2008).

Previous research has provided insights into how DRC settings influence perceived music quality among hearing aid users. Croghan et al. (2014) reported that listeners generally preferred linear amplification or slow-acting DRC over fast-acting settings, particularly highlighting that fast compression adversely affected music preference ratings due to distortions in both temporal and spectral features. Similarly, Madsen et al. (2015) found that clarity ratings for individual musical instruments decreased under DRC conditions compared to linear amplification, although no significant overall difference between fast and slow compression speeds was observed. However, considerable individual variability was evident, with some participants favoring slow-acting compression, potentially due to less alteration of temporal envelope shapes and spectral contrasts. These findings suggest that slow-acting compression may offer perceptual advantages for music listening, though listener preferences appear to be influenced by individual auditory profiles and the acoustic characteristics of the music.

Despite these contributions, several limitations remain. For instance, Madsen et al. (2015) assessed isolated instruments rather than full musical mixtures, which is well-suited for understanding source-specific clarity but does not address overall sound quality perception or additional perceptual dimensions such as sharpness. Furthermore, both Croghan et al. (2014) and Madsen et al. (2015) employed relatively short musical excerpts, included a limited range of genres, and relied on modest sample sizes. Both studies also relied on simulated hearing aids, which, while offering experimental control, may not fully capture the complexities of real-world hearing aid usage. Neither study examined the extent to which fast versus slow DRC settings compare directly to unaided listening, a comparison that is particularly relevant in light of reports that some hearing aid users prefer to listen to music without amplification (e.g., Greasley et al., 2020; Vaisberg et al., 2019). Moreover, both studies focused exclusively on subjective preference ratings, which provide valuable insight into listener impressions but offer limited information about the functional consequences of DRC settings for selective auditory processing.

### Auditory Scene Analysis in Music

A central focus in hearing aid research relates to the “cocktail party problem,” which refers to the ability to disentangle concurrent sounds that overlap in time and frequency, for example, separating a single voice from background noise and interfering speakers (McDermott, 2009). This ability is rooted in the process by which listeners organize complex acoustic environments into meaningful auditory objects and streams, a process known as Auditory Scene Analysis (ASA; Bregman, 1990). In the context of multi-source music, ASA allows listeners to segregate simultaneously occurring musical sounds, such as a solo oboe melody within an orchestra or a tenor voice in a choir. This application of ASA in the context of music is known as musical scene analysis (MSA; Bregman, 1990). Crucially, this perceptual process is not merely a supportive mechanism but enables the emergence of core musical structures such as melody and harmony in the first place (Huron, 2001). Accordingly, MSA underpins the very organization and perception of musical structure and is fundamental for the experience of musical textural layering, forms a perceptual foundation for musical clarity, and the ability to selectively attend to specific musical elements within a musical piece (Bey & McAdams, 2002; Huron, 2001).

For normal-hearing individuals, MSA functions seemingly effortless, allowing rich musical experiences where distinct instruments and melodies may be appreciated individually and as a harmonious whole. Hearing impairments, such as sensorineural hearing loss, disrupt this process by compromising abilities like frequency resolution, temporal processing, and spatial hearing (Akeroyd, 2008; Middlebrooks, 2017; Moore, 2007; Phillips et al. 2000), which are all crucial for MSA. As a result, the rich, layered structure of music may collapse into a rather undifferentiated auditory percept, diminishing clarity and blurring timbral contrasts. This may negatively impact both perceived sound quality and musical enjoyment. Tinnitus, a condition characterized by the perception of sound without external stimuli (Møller, 2006), has been reported to further impair ASA abilities beyond those caused by hearing loss (Durai et al., 2018, 2019).

Recent empirical work supports the role of MSA in shaping the perceptual experience of music. For example, Benjamin and Siedenburg (2024) demonstrated that hearing-impaired listeners with higher MSA performance tend to rate the sound quality of musical excerpts more favorably, suggesting that the capacity to hear out individual instruments in musical mixtures may enhance the esthetic evaluation of the music and potentially listener satisfaction. Nonetheless, the relationship between MSA and perceived sound quality may not be entirely linear. In a survey of hearing aid users, Madsen and Moore (2014) found that although approximately one-third of respondents reported improved clarity of musical instruments when using dedicated music programs in their hearing aids, no consistent differences in overall tonal quality were reported. This suggests a more complex and potentially listener-specific interaction between ASA processes and subjective sound quality evaluations. Taken together, these findings underscore the need to assess MSA abilities behaviorally, alongside subjective ratings, to gain a more comprehensive understanding of how hearing aid processing strategies affect music perception in ecologically valid listening contexts, particularly those involving complex, dynamically interacting sound sources.

Numerous tests have been developed to quantify (hearing-impaired) listeners’ ASA abilities in the speech domain, including the Oldenburg sentence test by Wagener et al. (1999) and the Göttingen sentence test (Kollmeier & Wesselkamp, 1997). In contrast, while several perceptual tasks exist for assessing specific aspects of music perception, such as mistuning detection (Larrouy-Maestri et al., 2019; Madsen & Oxenham, 2024), beat perception (Harrison & Müllensiefen, 2018), or melody discrimination (Harrison et al., 2017), very few probed MSA processes specifically. Even those studies that address MSA processes have typically been developed and validated exclusively with normal-hearing populations only (Susini et al., 2020). The “Adaptive Music Perception” test by Kirchberger and Russo (2015) constitutes a notable exception; however, the subtest related to MSA yielded inconclusive results, with some hearing-impaired participants unable to complete the task successfully. To address this gap, Hake et al. (2024) introduced a new adaptive MSA paradigm designed to efficiently assess MSA abilities—including among hearing-impaired individuals—by requiring detection of target instruments embedded in recorded polyphonic mixtures from diverse musical genres. Nevertheless, this paradigm has not yet been applied to individuals using hearing aids, leaving open important questions about the influence of amplification and signal processing strategies on MSA performance.

### The Current Study

The present study investigates the impact of hearing aid amplification and DRC settings on both MSA abilities and perceived sound quality among hearing aid users. Specifically, this research aims to evaluate how different hearing aid conditions—fast DRC settings and slow DRC settings—affect the ability to analyze musical scenes and the subjective quality of musical excerpts compared to that of not wearing hearing aids. To ensure ecological validity, all auditory stimuli were drawn from professionally recorded multitrack music. As a complementary measure, speech reception thresholds (SRTs) were included to investigate how individual differences in speech-in-noise intelligibility relate to MSA ability and sound quality ratings. Overall, amplification by hearing aids is expected to outperform the unaided condition for SRT. Although hearing aid users often report mixed experiences with music listening, a similar improvement is anticipated for MSA abilities and reported musical sound quality. We further hypothesize that hearing aids with fast DRC settings yield the best performance in MSA and SRT, while slow compression, in contrast, result in higher sound quality ratings compared to fast DRC settings (see Croghan et al., 2014; Moore, 2008).

## Methods

### Participants

**Figure 1.**
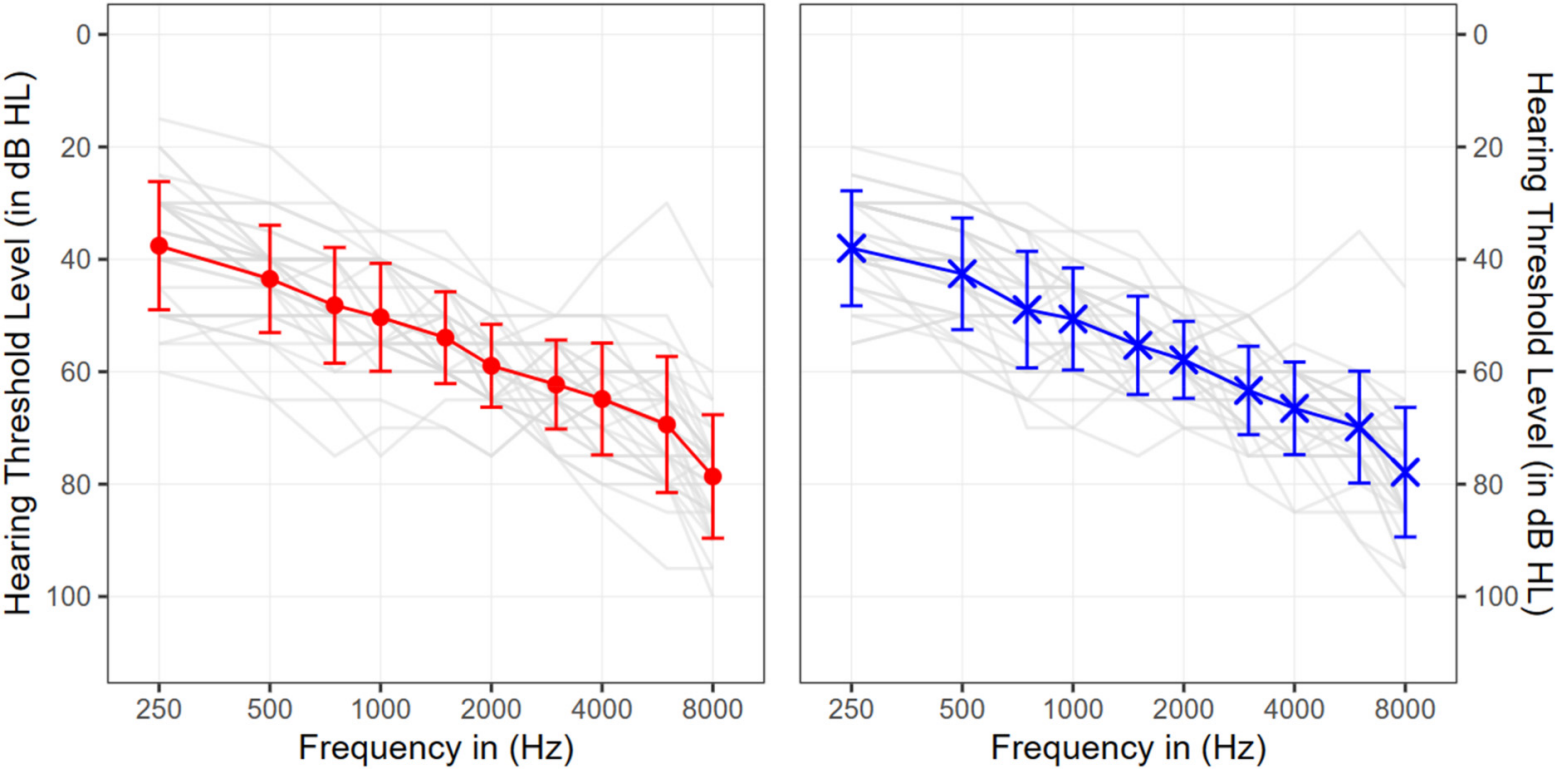
Average pure–tone audiometric threshold (PTA) measurements across participants for both ears (red = right ear; blue = left ear). The gray lines show individual participants' threshold. The bold solid lines are representing the pure–tone average over all participants for each listening group.

This study involved 33 participants with moderate to severe sensorineural hearing loss (*M*_age_ = 73.9 years, *SD* = 10.7 years; 31–78 years range; 17 female). The average duration of hearing aid experience among participants was 18 years and 10 months (*SD* = 10.8 years; 5–54 years range). To ensure a rather homogeneous sample, participants were required to have used their hearing aids consistently (at least 4–8 hr a day) for at least six months and to have a hearing loss classified within the N3(moderate)-N4(moderate to severe) range according to the Bisgaard profile (Bisgaard et al., 2010). Asymmetrical hearing loss was required not to exceed 20 dB at audiometric frequencies up to 8 kHz and an air-bone gap was not to exceed 15 dB at frequencies up to 4 kHz, with a single exception permitted outside this range. Individuals with active ear disease in one or both ears, contraindications to traditional hearing devices (i.e., anatomical anomalies, or other health-related factors that render the use of conventional air-conduction hearing aids inadvisable, inappropriate, or potentially harmful for a patient), or medical conditions requiring clearance for hearing aid fitting (e.g., mastoid cavity) were excluded. Individual hearing losses are illustrated in Figure 1.

### Materials

#### Audiometry

Hearing sensitivity was assessed in 5 dB HL steps using a PC-controlled clinical Madsen Astera audiometer (Firmware version 1.5.18). Pure-tone thresholds were measured at .125, .25, .5, 1, 2, 4, and 8 kHz. Given the significance of elevated hearing thresholds at frequencies above 4 kHz for complex ASA listening scenarios (Moore, 2020; Narne et al., 2023) pure-tone-average (PTA) was calculated across all measured frequencies.

#### Musical Scene Analysis Test

The adaptive MSA test (Hake et al., 2023) is a listening task developed to assess participants’ selective listening abilities for realistic music stimuli. Stimuli are sourced from an open-source music-database (“MedleyDB,” Bittner et al., 2014, 2016), which consists of real-world multitrack music recordings representing a wide range of musical genres (e.g., pop, rock, world/folk, fusion, jazz, rap, classical). The MSA adopts a “yes–no” paradigm that resembles a 2-alternative-forced-choice task. In each trial, participants listen to an 8-s excerpt of the full musical piece, containing all the instruments and voices included in the original recording. This is followed immediately by a 2-s excerpt of a single instrument or voice (the target), a 1-s silence, and finally a 2-s multi-instrument excerpt (the mixture). Participants had then to decide whether the target was part of the mixture or not. The 8-s presentation of the full musical piece provided sufficient time for the hearing aid's compression system to adapt to the acoustic environment. The task was varied in difficulty by manipulating three conditions: (a) the number of instruments in the final mixture (three or six instruments), (b) the choice of the target instrument (bass, guitar, lead-vocals, and piano), and (c) the target-to-mixture level ratio (ranged from −15 to 0 dB). All MSA stimuli were presented monaurally and without spatial separation of sources. The test initiates with a randomly selected excerpt of medium difficulty. Subsequently, the test adapts to the listener's performance, becoming progressively more challenging or easier as appropriate. This approach enables a reliable estimation of the participant's ability level with a short testing time. The standard test length of 30 items was applied. Participant ability is estimated using weighted-likelihood methods on a scale of −4 to +4; higher scores signify better performance. A score of 0 indicates performance equal to the median of the reference sample (*SD* = 1), which predominantly incorporated normal-hearing individuals (see Hake et al., 2023). The musical pieces contained in this database are, in general, not widely known to the public. A complete list of the excerpts used, along with the source code of the test, is publicly available (see github.com/rhake14/MSA). The current study employed MSA (long) Version 2.8.3.

#### Sound Quality Rating Task

Participants were asked to evaluate the quality of thirty 30-s musical excerpts drawn from five pieces of both pop and classical genres. To ensure comparability with the stimuli used in the MSA task, excerpts from the pop genre were sourced from the MedleyDB (i.e., the same database as used in the MSA task). In contrast, classical excerpts were drawn from commercial recordings and included works by Beethoven, Haydn, Mendelssohn, Mozart, and Salieri. These compositions were selected to represent well-known repertoire with diverse orchestration, providing a comprehensive basis for evaluating hearing aid processing strategies across a range of acoustic and structural features. A complete list of all musical excerpts used in both tasks is provided in Table A1 in the Supplementary Materials (hereafter referred to as “Supp. Mat.”). Each excerpt's sound level was normalized in root mean square (RMS). Quality ratings were conducted across five dimensions, adapted from the “Dimensions of Quality Ratings for Music” questionnaire by Davies-Venn et al. (2007; see also Gabrielsson et al., 1988), using a 10-point Likert scale with no neutral option; higher values indicated a more pronounced intensity of the respective dimension. The dimensions evaluated were loudness (“*How loud was the sound?*,” with very weak/faint to very loud), sharpness (“*How sharp/shrill or soft was the sound?*,” very soft to very sharp), fullness (“*How full was the sound?*,” very thin to very full), overall quality (“*How good was the overall sound quality of the music?*,” bad to good), and clearness (“*How clearly were you able to distinguish between the individual instruments?*,” unclear to very clear). These quality rating dimensions have been validated for the use with elderly hearing aid wearers (Narendran & Humes, 2003). Each dimension was presented in German, translations are provided in Table A2 in “Supp. Mat.”

#### Speech Reception Thresholds

Participants’ speech reception thresholds were measured using the Göttingen Sentence Test (GÖSA; Kollmeier & Wesselkamp, 1997). The GÖSA is a speech intelligibility in noise test that employs a standard adaptive procedure, where the masker level remains constant and the speech level is adjusted to determine the 50% speech reception threshold. The test includes 20 everyday sentences per list selected from a pool of 200 sentences, each containing three to seven words, spoken by a male speaker and embedded in speech-shaped white noise. Lower SRT scores (expressed in dB) indicate better performance.

#### Musical Training

To evaluate the individual's level of musical training, the German version of the Goldsmith Musical Sophistication Index (GMSI; Müllensiefen et al., 2014) was employed. The GMSI is a brief self-report questionnaire designed to assess various aspects of musical expertise, including subscales for active engagement and perceptual abilities. For this experiment, only the subscore for musical training was used, which comprises seven items. Participants responded to questions on a 7-point Likert scale (e.g., `I engaged inregular, daily practice of a musical instrument (including voice) for_ years.', with 0 / 1 / 2 / 3 / 4–5 / 6–9 / 10 or more; `I have never been complimented for my talents as a musical performer' with 1 = Completely Disagree to 7 = Completely Agree [reverse coded]). The final composite score ranges from 1 to 7, with higher scores indicating higher level of musical training.

### Procedure

This study was conducted between February and April 2024. Participants were recruited by Hörzentrum gGmbH. The experiment was administered using MATLAB and *psychtestR* (Harrison, 2020), an R package for creating web-browser-based behavioral experiments. All instructions were presented in German. Participants had prior experience with auditory experiments and were compensated on an hourly basis. Following an initial audiogram measurement using a standard clinical ascending-descending procedure, a total of three hearing test were conducted: First, three sets of the adaptive MSA test (Hake et al., 2023) were performed (30 items per set), each presented under a different hearing aid condition: (a) without hearing aids, (b) with hearing aids utilizing fast DRC settings, and (c) with hearing aids utilizing slow DRC settings. The second assessment involved musical sound quality ratings (SQR), evaluating the perceived sound quality of 30-s excerpts drawn from five pop and five classical music pieces. Pop samples were drawn from the same music database as the items used in the MSA task. Classical excerpts were sourced from openly accessible recordings (see Table A1 in Supp. Mat. for a complete list). A total of 30 items was presented, with three items extracted from each music piece and assigned to one of the hearing aid conditions. The third test measured speech intelligibility in noise (SRT)—likewise conducted three times, in each hearing aid condition, respectively. All tests were presented via a single frontally positioned loudspeaker at a long-term sound level of 70 dB SPL(A). The order of conditions and the specific stimuli presented in each condition were counterbalanced across participants. In addition, the aided conditions were blind to the listeners, thereby minimizing potential expectation biases. Importantly, participants completed structured training rounds for each of the three listening tasks during the experimental session (i.e., on the same day) to ensure understanding of the procedures prior to the actual data collection. Specifically, for the sound quality assessment, participants were provided with definitions of each quality attribute, accompanied by example stimuli that were artificially manipulated to represent exaggerated instances of each perceptual dimension (clearness, sharpness, etc.). This training procedure was implemented to ensure that participants correctly understood the intended interpretation of the rating scales, minimized variability arising from inconsistent internal standards, and were thereby able to make appropriate and informed judgements during the rating task. For the SRT and MSA tests, participants practiced with separate training items and could repeat the training upon request (minimum of seven items). During the training phase, participants were asked to confirm the audibility of target signals to ensure that stimuli were perceptually accessible before proceeding to the experimental tasks. Finally, participants completed the standard demographics questions and the musical training subscale of the Goldsmith-Musical Sophistication Index (Müllensiefen et al., 2014). Participants were encouraged to take breaks between tests, and additional breaks were offered as needed. In total, data collection was completed in a single session, with scheduled breaks after each full task and additional breaks provided upon request. The session lasted approximately two hours. This study was approved by the Carl von Ossietzky University Oldenburg ethics review board (Drs.EK/2021/031-05), and all standard practices, including informed consent, were followed prior to data collection.

#### Hearing Aid Fitting

Participants were binaurally fitted with target fitting software for Audéo L90-R receiver in the canal hearing aids from Phonak. Amplification was defined based on a NAL-NL2 fitting rationale and individual hearing thresholds. The choice of NAL-NL2 was motivated by the amount of prescribed compression necessary to provide effective differences between the tested conditions for the given range of hearing losses. Acoustical coupling was made with standard power domes to maximize the ratio between amplified and direct sound. Frequency lowering was disabled as well as all the noise cleaning algorithms as they are mainly designed for clean speech or speech-in-noise listening situations. Microphone modes were set to ‘Real Ear Sound’, a beamformer pattern close to the natural pinna effect. Critical gain was measured individually, and feedback canceller was left on the default value. The DRC modes were aligned with those outlined by Chen et al. (2021), where the fast-acting compressor operated with an attack time of 10 ms and a release time of 60 ms, and the slow-acting compressor used an attack time of 1.2 s and a release time of 7.1 s.

### Analysis

Statistical analyses were conducted using both MATLAB and R statistical software (v.2022.07.2 + 576; RStudio, 2020). Mixed effects models, including random effects for participants, were employed, and contrasts were used to mitigate the risk of *p*-value inflation (alpha is set to .05). Effect sizes and confidence intervals, in addition to *p*-values, were reported to provide a comprehensive understanding of the results. Correlational analyses were conducted using Pearson's correlation for continuous data and Spearman's rank-order correlation for relationships between continuous and ordinal data. Likelihood ratio tests were applied to compare nested models and assess whether the inclusion of additional predictors significantly improved model fit. Data point exclusion was based on Cook's distance. Specifically, three data points, which were spatially separated from the others (see Figure A1 in “Supp. Mat.”), showed exceptionally poor performances close to chance level. Consequently, these data points were replaced using multiple imputation by chained equations with the *mice* package (v.3.16.0) in R. Specifically, pooled estimates using Bayesian linear regression method with PTA, SRTs, and quality ratings were used to predict MSA scores across five datasets (over 50 iterations). This procedure accounts for variability and uncertainty in test scores, ensuring robust imputation estimates.

## Results

### Effect of Amplification and Compression Across Listening Tasks

#### Musical Scene Analysis Abilities

**Figure 2.**
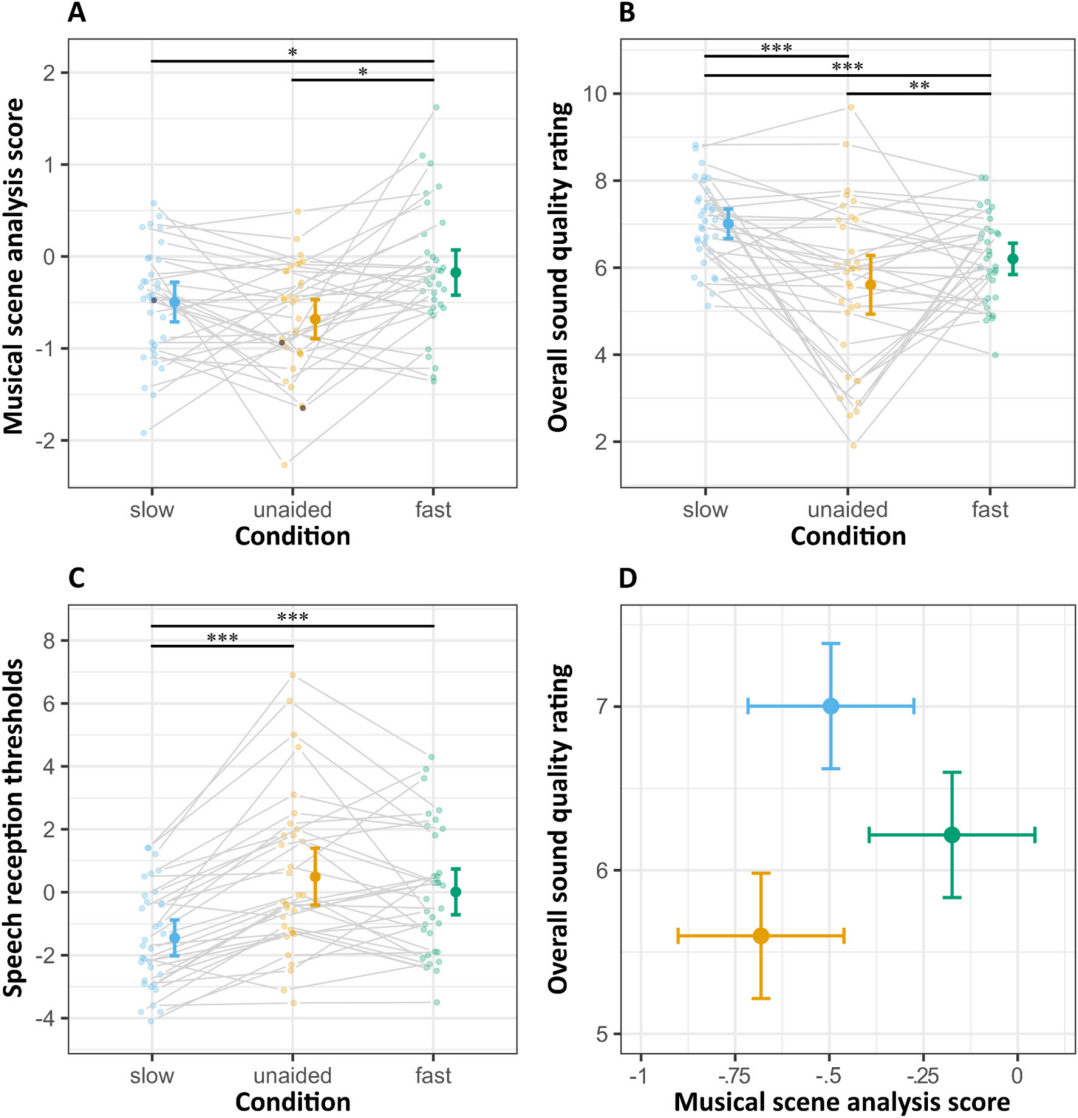
Differences in mean (A) musical scene analysis (MSA) scores, (B) overall sound quality rating (SQR) scores, and (C) speech reception thresholds (SRT in dB SNR) across different conditions: slow (blue), unaided (yellow), fast (green). Error bars represent 95% confidence intervals. Individual participant data are displayed as shaded dots. Panel D shows the estimated marginal means and the relationship between MSA scores and SQR scores for each condition (comparisons between SQR and SRT and SRT and MSA can be found in Figure A2 in Sup. Mat.). Model summaries can be found in Table A4, A6, and A7 in Sup. Mat.

*Note*. Significance at *p < .05, **p < .01, ***p < .001.

MSA abilities were found to be lowest in the unaided condition, which served as the reference (*M* = −.68, *SD* = .60; see Figure 2A). When comparing the compression approaches, the fast DRC condition (*M* = −.17, *SD* = .61) outperformed the slow DRC condition (*M* = −.50, *SD* = .69). Notably, when listeners were equipped with the DRC setting that yielded the most beneficial outcome in the MSA task, the average MSA ability estimate reached near-normal levels (*M* = .003, *SD* = .7). A mixed effects model (Model A1) was fitted to predict MSA scores based on listening conditions (unaided, fast DRC, and slow DRC). The counterbalancing of listening condition presentations across participants was not entirely successful, with one order being represented five times more frequently than the others. To address potential order effects, two additional models were fitted, incorporating the order of presentation and its interaction with listening conditions (Models A2 and A3, respectively). All models included random effects for participants and fixed effects for listening conditions, in which the intercept was allowed to vary. The effect of order was found to be non-significant (*β* = −.07, *p* = .36). A likelihood ratio test further indicated that including order did not significantly improve model fit, *χ*²(1) = .88, *p* = .35, nor did including the interaction of order (see Table A3 in Supp. Mat.). Likewise, adding musical training as a covariate (Model A5) did not improve model fit for the MSA performance data, *χ*²(1) = .03, *p* = .87, and was therefore not retained. Accordingly, Model A1 was used for the analysis.

The effect for amplification was estimated by comparing the unaided condition with the average of the aided conditions under fast and slow DRC settings, while compression effects were evaluated by directly comparing the fast and slow DRC settings. The results indicated significant effects for amplification (*β* = −.43, *SE* = .16, *p* = .01, *d* = .63), indicating that the aided conditions showed a significant improvement over the unaided conditions, with a moderate effect size. Furthermore, an effect for compression was found (*β* = −.43, *SE* = .19, *p* = .03, *d* = .51), with fast DRC showing improved performance over slow DRC settings, with an effect size approaching moderate significance. Overall, there is considerable individual variability in MSA scores, as indicated by the random effects, with a variance of .10 and a standard deviation of .32. No significant correlation was found between unaided MSA performance and better-ear PTA, *r*(31)= −.28, *p* = .12.

Thirteen out of thirty-three participants (39%) reported experiencing tinnitus lasting longer than three months; however, mixed-effects analyses revealed no significant effect of tinnitus on performance (or in ratings respectively) in any of the tasks, including MSA, SRT, or SQR (see Tables A3 and A11 in the Sup. Mat.). Consequently, tinnitus status was not considered in subsequent analyses.

#### Sound Quality Ratings

To evaluate overall sound quality, the “overall quality” item was used as the primary measure. Overall quality was highly correlated with clearness (*r*(988) = .69, *p* < .001), and fullness (*r*(988) = .59, *p* < .001). Moderate correlations were observed between overall quality and loudness, (*r*(988) = .44, *p* < .001). In contrast, sharpness showed a lower yet still significant correlation with overall quality with a Pearson correlation (*r*(988) = .2, *p* < .001). These results are presented in detail in the correlation matrix Table A5 and Figure A3 (see Supp. Mat.).

Mixed-effects models were fitted to predict the overall sound quality scores (SQR) based on listening condition (unaided, slow, fast) and musical genre (classical vs. pop) to assess whether adding musical genre, as well as the interaction between condition and genre, revealed meaningful effects or improved the model fit. Four models were compared to determine the most suitable fixed and random effects structure for the data. The first model (B1) included only the listening condition as a fixed effect, while the second model (B2) included both the listening condition and musical genre as fixed effects. The third model (B3) further incorporated the interaction between listening condition and musical genre as a fixed effect. All three models (B1–B3) included random intercepts for both participants and stimuli. In the fourth model (B4), the random slope for listening condition was allowed to vary by stimulus; however, this model encountered boundary issues, leading to a singular fit, and was consequently discarded. Adding musical training as a covariate did not improve model fit for the subjective sound quality ratings, *χ*²(1) = .03, *p* = .87, and was therefore not retained. Model comparison indicated that the second model (B2), which included listening condition and musical genre as fixed effects, provided the best fit. Detailed model comparison is available in Table A3 (Supp. Mat.).

Overall, the mean quality rating for the slow condition was the highest at 7 (*SD* = 1.45), followed by the fast condition at 6.22 (*SD* = 1.67), and the unaided condition had the lowest mean rating at 5.6 (*SD* = 2.24; see Figure 2B). Using contrast specifications similar to the analysis conducted for the MSA scores, an effect for hearing aid amplification was found, indicating that the average of both aided conditions was rated higher than the unaided condition, with (*β* = 1.01, *SE* = .1, *p* < .001, *d* = .66). This effect size reflects a substantial improvement in perceived sound quality. Further contrast analysis revealed a significant compression effect (slow vs. fast DRC), with the slow condition rated higher than the fast condition (*β* = .514, *SE* = .1, *p* < .001, *d* = .51), indicating a clear preference for the slow condition with a moderate effect size. Similar to the MSA, SQR scores exhibit substantial individual variability, with the random effects model indicating a variance of .87 (*SD* = .93) across participants.

**Figure 3.**
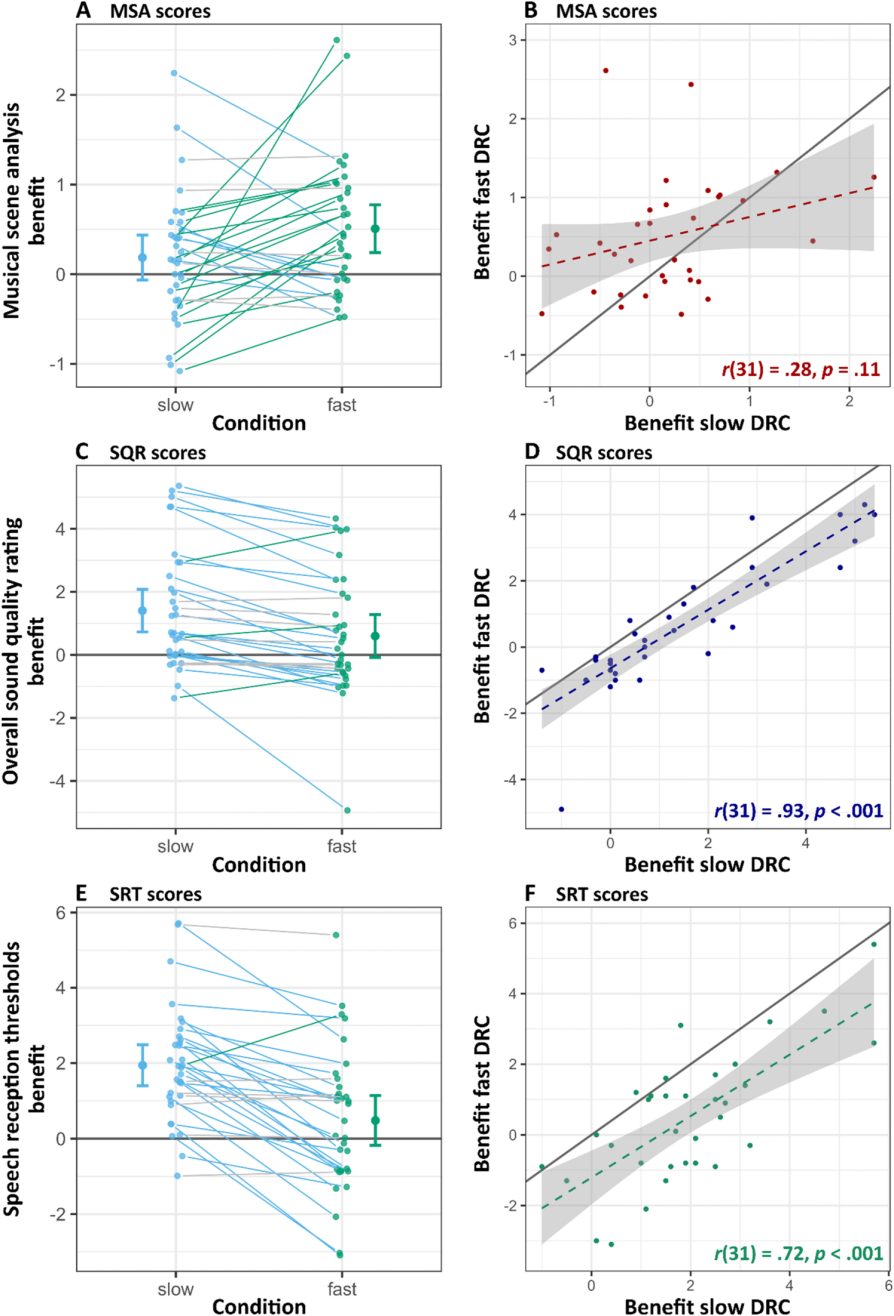
Effects of hearing aid compression settings on musical scene analysis (MSA), overall sound quality rating (SQR), and speech reception threshold (SRT) scores. Panel A, C, and E show individual benefit scores (that is the unaided condition was either subtracted from the slow or fast DRC condition) of the MSA, SQR, and SRT, respectively. Solid lines connect individual participant scores under DRC conditions, with green lines representing participants who performed better with fast DRC, blue lines for better performance with slow DRC, and grey lines indicating indistinct differences in performance (within ±10% of one standard deviation of the respective benefit distribution). Panels B, D, and F show scatterplots for benefit scores from slow and fast DRC for all tasks. The dashed lines are linear regression fits with 95% confidence intervals shaded in grey, indicating the trend and variability for each test. The solid black diagonal line in each of these panels represents the line of equivalence, where points along this line would indicate equal benefit from fast and slow DRC settings. Pearson correlation coefficients are reported in the lower right corner.

There were also differences in variability across excerpts; however, compared to the subject-specific individual differences, this variability was relatively low, with a variance of only .09 (*SD* = .29). When examining stimulus-specific effects, aided listening conditions generally showed improvements in sound quality perceptions, with slow DRC often outperforming fast DRC, yet some stimuli received similar ratings across all conditions, indicating minimal impact of processing (see Figure 3). Notably, in certain cases, unaided listening was even preferred over fast DRC (i.e., unaided received higher SQR), suggesting that the benefits of DRC may be stimulus-dependent. The model identified a significant main effect of genre, with pop excerpts receiving less favorable ratings (*M* = 6.11, *SD* = 1.97) compared to classical excerpts (*M* = 6.44, *SD* = 1.82). This main effect was statistically significant (*β* = −.33, *SE* = .15, *p* = .023) with a small effect size (*d* = .22). The interaction between genre and condition was non-significant (χ²(2) = 3.02, *p* = .221) and the increase in model fit due to the interaction term was also minimal, with the marginal R² increasing only from .358 to .36. For a detailed descriptive comparison between genres and sound quality attributes, see Figure A4 and A5 (in Supp. Mat.). Unaided sound quality ratings showed a significant negative correlation with better-ear PTA (*r*(31)= −.42, *p* = .02).

#### Speech Reception Thresholds

The results showed that the unaided condition had the poorest speech-in-noise performance, with the speech reception thresholds (SRT) scoring highest (*M* = .49, *SD* = 2.55; see Figure 2C), followed by the fast DRC condition (*M* = .01, *SD* = 2.05) and the slow DRC condition (*M* = −1.45, *SD* = 1.61). Following the procedure used for MSA and SQR, several mixed effects model were fitted to predict SRT scores and examine the effects of hearing aid amplification and compression. Similarly to the other tasks, neither including genre (Model C2), nor musical training (Model C3) as a covariate for SRT did not improve model fit and were therefore not retained (see Table A3 in Supp. Mat for details). Listening conditions were included as a fixed effect, and random effects allowed the intercept to vary by participants. Contrasts analysis of the final model (C1) revealed significant effects for amplification, with aided conditions (fast and slow DRC) showing improved SRT scores compared to the unaided condition (*β* = −1.21, *SE* = .24, *p* < .0001, *d* = 1.08), reflecting a large effect size. A significant compression effect was also observed, with the slow DRC condition outperforming the fast DRC condition (*β* = 1.46, *p* < .0001, *d* = .43), indicating a moderate effect size. A significant positive correlation emerged between unaided SRT scores and better-ear PTA (*r*(31) = .72, *p* < . 001).

### Amplification Benefits Across Individuals and Listening Tasks

The impact of DRC settings on different listening tasks was further explored by examining individual participant performance across tasks. First, a set of linear mixed-effects regression models was conducted in which each outcome variable was predicted by the remaining two, to examine whether individuals who performed well in one task also tended to perform well in others. All models included random intercepts for participant and listening condition. The results revealed significant associations between behavioral outcomes: When predicting SRT, both MSA (β = –0.42, SE = 0.17, *p* = .016) and SQR (β = –0.24, SE = 0.11, *p* = .034) emerged as significant predictors, indicating that individuals with better MSA performance and more favorable quality ratings also showed improved speech intelligibility (i.e., lower SRTs). In the reverse model, however, only SRT significantly predicted SQR (β = –0.22, SE = 0.07, *p* = .003), whereas MSA did not (β = 0.11, SE = 0.16, *p* = .508). Finally, when predicting MSA scores, SRT was a significant predictor (β = –0.14, SE = 0.04, *p* = .002), while SQR was not (β = 0.03, SE = 0.06, *p* = .579). These findings suggest that, on average, that individuals who performed well in one auditory task tended to perform better in others; however, these associations were not consistent across all tasks, indicating only partial overlap in performance across domains. Full model summaries are reported in Tables A8–A10 in the Supp. Mat.

Furthermore, to evaluate the benefit of amplification across DRC settings, the individual relative improvement in performance between aided and unaided listening conditions was determined for each task. As shown in Figure 3 the effects of DRC on MSA, SQR, and SRT varied across individuals. For SQR and SRT, a strong positive correlation was observed between the benefits from slow and fast compression settings, *r*(31) = .93, *p* < .001, and *r*(31) = .72, *p* < .001, respectively. This pattern suggests that individuals who benefited from one DRC condition tended to benefit from the other, albeit to varying degrees (see Model B2 and C1). More specifically, 23 individuals rated the overall sound quality (i.e., SQR) higher with slow DRC (i.e., individual dots falling below the line of equivalence in Figure 3B), while only three rated fast DRC higher (for an overview over preference for the other quality dimensions see Figure A6 in Sup. Mat.). Seven individuals showed only minor differences between the two conditions, that is within a ±10% margin of 1*SD* benefit distribution, indicating no clear advantage for one DRC setting over the other (see Figure 3C for individual trajectories). A similar pattern emerged for SRT, with 25 individuals performing better with slow DRC, one performing better with fast DRC, and seven showing indistinct differences (Figure 3E). In contrast, for MSA, the benefits from slow and fast DRC were less consistent across individuals. Nine participants performed better with slow DRC, 18 with fast DRC, and six showed indistinct differences (Figure 3A) and the correlation between slow and fast compression settings for MSA was weak and non-significant, *r*(31) = .28, *p* = .11 (Figure 3B). This highlights that the impact of DRC on MSA varies considerably across individuals compared to SQR and SRT. Although, on average, fast DRC yielded higher scores for MSA, several individuals demonstrated better performance with slow DRC, underscoring the variability in how DRC settings affect MSA across participants.

**Figure 4.**
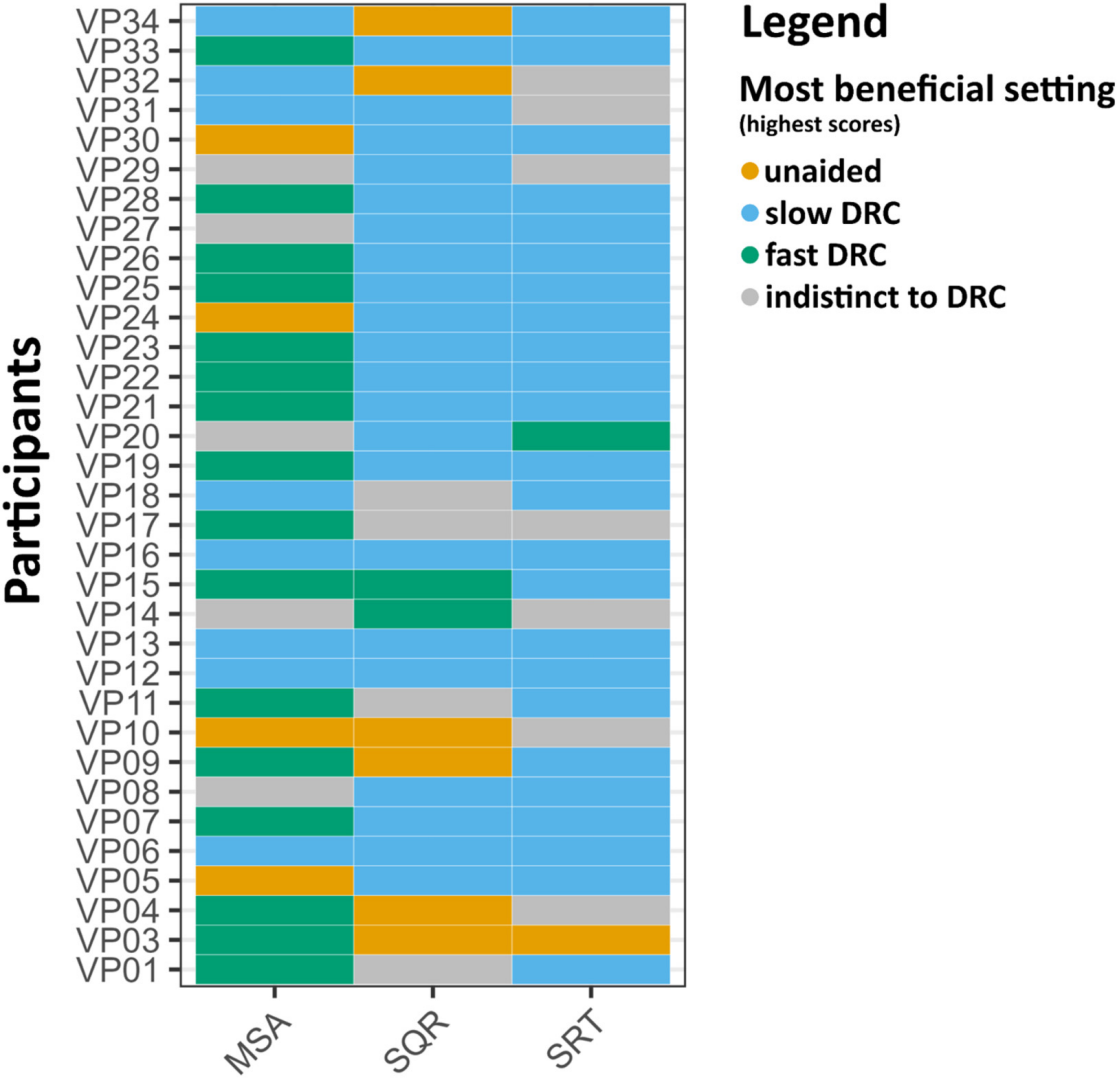
Most beneficial settings (heatmap) for all three conditions (unaided vs. aided fast DRC vs. aided slow DRC) across the three tasks: Musical Scene Analysis test (MSA), overall Sound Quality Ratings (SQR), and Speech Reception Thresholds (SRT). Each row represents a participant, and the columns correspond to the respective tasks. Colours indicate the hearing aid setting associated with the highest scores for each task: pale red (unaided), blue (slow dynamic range compression [DRC]), and green (fast DRC). Grey indicates indifference, where scores fell within a ±10% margin of one standard deviation of the respective benefit distribution, suggesting no clear advantage for any specific condition.

In addition to the differences observed in DRC effects within individual tasks, there was considerable variability in how participants benefited from amplification across tasks. Among all listeners, only four consistently showed the best outcomes across all tasks in the same hearing aid condition (hereafter referred to as individual “most beneficial setting”), specifically performing best with the slow DRC setting (see Figure 4). An exploratory check indicated that the four participants with consistent outcomes showed no notable commonalities in age, hearing loss severity, musical training, or hearing aid experience compared to the rest of the sample. The remaining participants exhibited task-specific differences, with the most beneficial hearing aid setting varying depending on the specific demands of each task. Notably, the strongest alignment was observed between sound quality ratings and speech reception thresholds, with 18 participants showing the highest scores in the slow DRC condition for both tasks.

To further examine the amplification benefit patterns across tasks, one total benefit score for each task (MSA, SQR, and SRT) was calculated. Specifically, for each task, the benefit of slow and fast DRC was calculated by subtracting the unaided score from the score in each DRC condition. These two values were then summed to yield a total benefit score for each task. Analysis of these total benefit scores revealed no significant correlations between benefits across tasks: the correlation between MSA total benefit and SQR total benefit was weak and non-significant, *r*(31) = .12, *p* = .51 (see Figures A7 in Supp. Mat.). Similarly, no significant relationship was found between MSA total benefit and SRT total benefit, *r*(31) = .13, *p* = .48, nor did the correlation between SQR and SRT total benefit scores reach significance, *r*(31) = .24, *p* = .19. When analyzed separately for each DRC setting, no significant correlations were identified across tasks in either the fast or slow DRC conditions (see Figure A8 in Supp. Mat.). However, a marginally significant relationship was observed between SRT and SQR benefits under the slow DRC condition, *r*(31) = .33, *p* = .06. This finding is consistent with the pattern observed in the heatmap (Figure 4), which shows that the majority of participants achieved their best results with the slow DRC condition in both the SRT and SQR tasks.

### Individual Differences in Hearing aid Amplification Benefit

**Figure 5.**
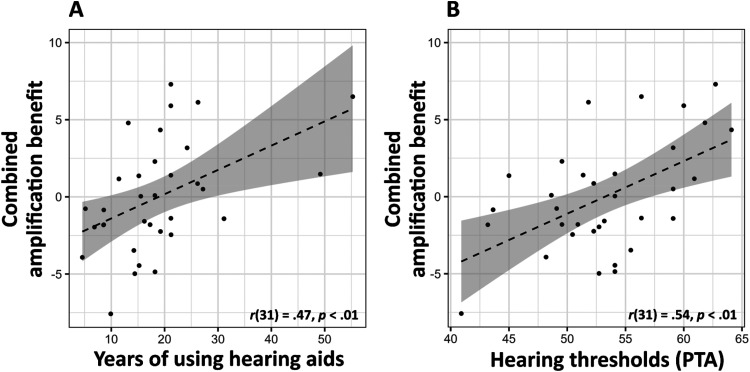
Scatterplots illustrating the relationship between individual characteristics and combined amplification benefit, a composite score reflecting the summed standardized benefit across all tasks (MSA, SQR, and SRT) under both DRC conditions. Panel (A) displays combined amplification benefit in relation to years of hearing aid use, and Panel (B) shows combined amplification benefit in relation to hearing thresholds (PTA). Pearson correlation coefficient is provided; the blue line represents the linear regression with a shaded 95% confidence interval.

To explore potential sources of individual variability in amplification benefit, associations were examined between a composite total amplification benefit score and individual factors, including musical training, age, and hearing aid use history. Each task-specific benefit score was first standardized, then summed across tasks to create a total amplification benefit score that reflects overall benefit from amplification. Total amplification benefit did not correlate with musical training (*r*(31) = −.19, *p* = .3) or age (*r*(31) = −.13, *p* = .47). Similarly, no significant group differences between males and females were found (*F*(1, 31) = 2.18, *p* = .15). There was a moderate positive relationship between years of hearing aid use and total benefit (*r*(31) = .45, *p* = .001), with greater benefit also associated with higher hearing thresholds (PTA) in the better ear (*r*(31) = .63, *p* < .001), suggesting that participants with longer hearing aid use and more severe hearing loss derived greater amplification benefits (see Figure 5).

These findings raise the question of whether the increased benefit is due to adaptation over time or simply reflects further hearing deterioration (i.e., higher PTA), which increases the contrast between aided and unaided conditions. To clarify these relationships, a linear regression model was conducted to assess the impact of years of hearing aid use and PTA on total benefit. The Variance Inflation Factor (*VIF* = 1.15) indicates that multicollinearity was not severe in this model. The regression analysis revealed that both years of hearing aid use (*β* = .12, *p* = .02) and PTA (*β* = .28, *p* < .001) were significant predictors of total amplification benefit. This suggests that both the duration of hearing aid use and the severity of hearing impairment contribute to the perceived benefit of amplification. The model accounted for 42% of the variance in total benefit (*R*^2^ = .42, *p* < .001), underscoring the importance of both factors. No interaction effect was observed between the two predictors (*p* = .89), indicating that the relationship between the duration of hearing aid use and total benefit does not depend on hearing threshold severity.

To further examine whether listener-specific factors influence the benefit of amplification, additional mixed effects models were conducted that included the following factors: better-ear PTA, age, years of hearing aid use, musical training, and gender. In the full model for the total benefit score (aggregated across tasks and DRC settings), both hearing loss severity (PTA: *β* = 0.22, *p* = .04) and years of hearing aid use (*β* = 0.11, *p* = .05) emerged as significant predictors, while age (*β* = –0.04, *p* = .46), musical training (*β* = –0.21, *p* = .70), and gender (*β* = 1.28, *p* = .26) were not significant.

Given that some listeners exhibited benefit primarily in either the fast or slow DRC condition, additional models were fitted separately for each DRC setting using composite standardized benefit scores across tasks. For the fast DRC benefit score, PTA showed only a marginal effect (*β* = .11, *p* = .08) and years of hearing aid use was marginal (*β* = .05, *p* = .09), while all other factors remained non-significant (age: *β* = –.01, *p* = .61; musical training: *β* = .07, *p* = .83; gender: *β* = .29, *p* = .64). For the slow DRC benefit score, PTA again showed a marginal effect (*β* = .12, *p* = .07), years of hearing aid use was marginal (*β* = .06, *p* = .09), and other predictors remained non-significant (age: *β* = –.02, *p* = .45; musical training: *β* = –.28, *p* = .39; gender: *β* = .99, *p* = .14).

### Simulated Hearing aid Output Target-to-Masker Ratio of the MSA Task

To better understand why fast-acting DRC outperformed the slow-acting DRC settings in the MSA task, the hearing aid output target-to-masker ratio (TMR) of the MSA test material (i.e., the excerpts) was computed by using the inversion technique (Hagerman & Olofsson, 2004). This procedure allows the separation of the hearing aid's output signal, allowing analysis of how each is processed by the device and to compute their respective levels. The principle is to record the same test signal twice: first with the target plus the mixture and then the target minus the mixture. Target and mixture at the output of the hearing aid are then extracted by adding and subtracting the recorded signals, respectively. This was done for all target instruments from the original pool of stimuli (that is, bass, guitar, lead-vocals, and piano). The reference hearing aid output TMRs were computed for a fitting with 0 dB insertion gain to have a comparable bandwidth with the aided conditions. Hearing aids were fitted with a NAL-NL2 fitting formula for a N4 hearing loss with the fast-acting and the slow-acting DRC.

**Figure 6.**
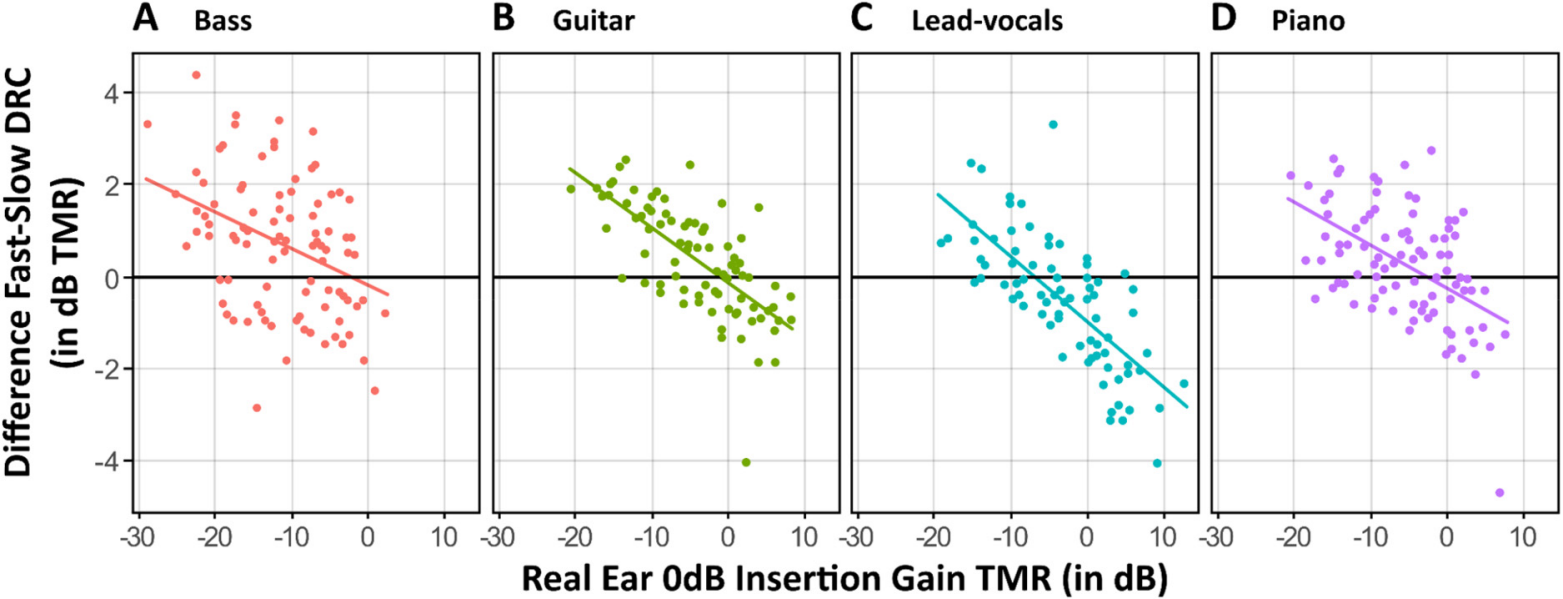
Difference in TMR between fast and slow DRC versus real ear TMR as reference for various musical instruments. The graphs show how the effect of compression varies with the reference TMR. Each subplot represents a different instrument: bass, guitar, lead-vocals, and piano. The vertical axis indicates the difference in TMR (dB) between fast and slow DRC where positive values are in favour of fast DRC, while the horizontal axis shows the real ear TMR (dB). The lines represent the linear regression fits for each instrument.

A consistent pattern was observed (see Figure 6): across all instruments, there is a negative correlation between reference TMR and the difference between fast and slow DRC TMR. The downward trend in each panel indicates that at negative reference TMRs (when the target instrument is more masked within the mixture), fast DRC provides greater benefit, yet the difference between fast and slow DRC settings gradually diminishes, meaning there's little benefit in using fast DRC over slow DRC. At higher reference TMRs (when the target instrument is more prominent in the mixture), slow DRC settings begin to provide more benefit. The average difference favored fast DRC for bass (.7 dB, 95% CI = [.4, 1]), guitar (.3 dB, 95% CI = [1, .6]), and piano (.3 dB, 95% CI = [.1, .5]), but slow DRC was more beneficial for lead vocals (−.5 dB, 95% CI = [−.8, −.2]).

## Discussion

The present study investigated the effects of hearing aid amplification on music and speech perception among individuals with moderate to severe hearing loss. Specifically, we examined how dynamic range compression (DRC) settings influence auditory outcomes by integrating behavioral assessments of MSA, musical sound quality ratings (SQR), and speech recognition thresholds (SRT). Unlike previous studies that primarily compared different types of hearing aid processing strategies, the present design directly contrasted unaided listening with two aided conditions (slow vs. fast DRC). This allowed for a delineation of amplification benefits per se, rather than only differences in signal processing techniques.

Overall, hearing aid use significantly improved scores across all three listening tasks (i.e., improved MSA and SQR scores and decreased SRTs) relative to unaided listening. Even if this may sound trivial, this result seems noteworthy in the music domain, where prior survey-based research has reported that a substantial proportion of hearing aid users tend to abstain from using their devices during music listening (e.g., Greasley et al., 2020; Vaisberg et al., 2019). Importantly, however, approximately one in four listeners either performed better or reported more favorable sound quality ratings in the unaided condition—an outcome that was particularly evident in the musical tasks. This finding underscores that amplification does not uniformly enhance auditory outcomes across individuals or perceptual domains and implies that the hearing aid settings applied may not be optimally suited to every user's perceptual needs or preferences. Practically, this may suggest the importance of including unaided conditions as a reference in clinical evaluation protocols. Moreover, it calls for greater transparency in communicating the potential variability of hearing aid benefit to users, as well as the need for context-sensitive fitting strategies.

### Task-Specific Effects of Compression Speed Benefit

The amplification effect elicited by the different DRC settings varied across tasks, revealing a nuanced interplay between compression settings and auditory outcomes. Our primary hypothesis was that slow DRC would lead to superior sound quality ratings due to its ability to preserve natural temporal envelopes and the dynamic range of sounds, thus reducing distortions and enhancing the listening experience (Moore, 2008). Conversely, fast compression was expected to improve the audibility of low-level sounds and assist in “listening in the dips,” thereby benefiting MSA (Siedenburg et al., 2020). The results aligned with these predictions: slow DRC elicited the highest subjective ratings of musical sound quality, while fast DRC yielded superior performance in the behavioral MSA task with a modest effect size though.

Furthermore, it was hypothesized that DRC effects in the speech domain would resemble those observed in music, given prior evidence indicating overlapping effects of hearing loss on both speech and music perception (e.g., Hake et al., 2023) and their reliance on shared auditory scene analysis (ASA) mechanisms. Specifically, previous research has demonstrated that fast-acting DRC can enhance speech perception in noise (e.g., Kowalewski et al., 2018). Supporting this assumption, a moderate correlation (*r* = .55) was observed between music- and speech-based ASA performance under unaided conditions, corroborating findings observed previously among normal-hearing individuals and non-hearing aid users (Hake et al., 2023). Interestingly, though, this relationship was no longer present under aided conditions. While the current study was not designed to directly test this dissociation, several explanations are plausible. One possibility is that DRC processing differentially alters the acoustic structure of speech and music, thereby disrupting shared perceptual mechanisms or unevenly altering task demands across domains. Moreover, aided listening introduces additional listener-specific variables—such as prior experience with hearing aids, personal preferences for compression speed, and degrees of acclimatization—that may further modulate perception in idiosyncratic ways. This added layer of inter-individual variability could further weaken the consistency of domain-general patterns, effectively eroding the correlation between musical and speech-related ASA performance in the aided condition.

Notably, the effects of DRC settings differed between the two perceptual domains. While fast-acting DRC yielded superior outcomes in the MSA task, slow DRC outperformed fast DRC in the speech intelligibility task, further highlighting the domain-specific nature of DRC-related perceptual effects in speech and music. Several potential explanations exist for this outcome. One possibility is that the differential benefits of slow versus fast DRC settings between speech and music arise from distinct—i.e., non-overlapping—aspects of auditory processing that are uniquely engaged by each domain. More specifically, music listening may engage different cognitive processing strategies compared to speech (in noise), potentially placing higher demands on attentional resources. However, there is substantial evidence of shared underlying cognitive processes involved in auditory perception across both speech and music domains (e.g., Barros et al., 2017; Shinn-Cunningham & Best, 2008). This overlap is so pronounced that Sallat and Jentschke (2015) have proposed using musical material as a tool for early diagnosis of speech-related impairments. Accordingly, the differing outcomes for MSA and SRT tasks may be attributed to the specific acoustic characteristics of each task instead. For example, compared to speech in noise, music—with its rapid fluctuations—more often contains short temporal dips that can benefit from rapid gain adjustments to maintain the audibility of all elements (Chasin, 2012; Siedenburg et al., 2020). Fast compression may capture these rapid fluctuations more effectively and thus better restore audibility of musical elements. At the same time, however, fast DRC can amplify noise components in speech gaps, making “listening in the dips” more difficult in speech-in-noise tasks (May et al., 2020). Furthermore, fast compression can introduce temporal distortions either by altering the natural modulations of the signal or by introducing fluctuations in the shape of the temporal envelope (Madsen & Moore, 2014; Stone & Moore, 2008). This trade-off likely contributed to the lower music sound quality ratings observed for fast DRC settings (see also Croghan et al., 2014; Moore, 2008). Conversely, slow DRC adjusts the gain gradually, preserving signal clarity and avoiding abrupt gain changes that could introduce artifacts, effectively maintaining the acoustic integrity of the sound. This contributes to a more natural and pleasant listening experience. Slow DRC also retains the natural dynamics of the speech envelope, enhancing speech intelligibility in steady-state noise environments where glimpses are less frequent. Notably, in environments with multiple sound sources, fast DRC has been shown to outperform slow DRC in enhancing speech intelligibility, likely by capturing auditory glimpses (“dip listening” opportunities), e.g., demonstrated by Salorio-Corbetto et al. (2020) in multi-talker scenarios. Similar results have been observed with negative test SNRs when applying fast-acting compression to speech in the presence of fluctuating, rather than with steady, background noise (Desloge et al., 2017; Rhebergen et al., 2009, 2017). Accordingly, the benefit of different DRC settings may depend on scene dynamics, that is, the modulation characteristics of both the masker and the target signal.

Previous studies have suggested that the benefit of slow versus fast DRC further depends on the signal-to-noise ratios (SNRs). Fast-acting compression may improve audibility at negative SNRs, whereas enhanced gain recovery can improve the audibility of soft speech segments that follow louder sounds. In contrast, at more favorable (positive) SNRs, where the speech signal is already dominant, fast DRC may degrade performance by amplifying background noise unnecessarily and distorting the natural temporal envelope of speech. In such contexts, slow-acting DRC typically offers better preservation of the temporal structure and spectral integrity of the acoustic signal (Jenstad & Souza, 2005; Stone & Moore, 2008). Our findings suggest a similar conditional benefit of DRC for music perception. Analyses of hearing aid output signals revealed that fast-acting DRC provided greater benefit compared to slow DRC primarily at negative target-to-mixture ratios (TMRs), where the target musical instruments were less audible within polyphonic mixtures. The rapid gain adjustments afforded by fast compression appeared to improve the audibility of low-level instrumental signals following louder passages, thereby supporting more effective MSA. However, as TMRs increased and the target became more salient, the relative advantage of fast DRC diminished, and slow DRC gained superiority. The behavioral data thus corroborate prior findings in speech tasks and extend them into the domain of music.

Nonetheless, critical differences in perceptual goals between music and speech listening contexts must be acknowledged. In speech perception, the objective typically centers on extracting a single intelligible voice, whereby competing sources are treated as distractors. In music, however, no such distractors exist; instead, the listener engages with multiple concurrently sounding instruments, including softer ones embedded within polyphonic textures. From a performance perspective, this supports the use of fast-acting DRC, which may enhance MSA by improving audibility across the entire dynamic range, allowing listeners better perceptual access to all musical elements. Indeed, behavioral data from the MSA task indicated that, on average, fast DRC resulted in the greatest improvement in performance and benefited the largest proportion of listeners. Yet, the subjective ratings revealed a divergent pattern. The majority of participants rated slow DRC more positively in terms of overall musical sound quality. Furthermore, slow DRC also received higher subjective ratings for the item “*How clearly were you able to distinguish between individual instruments?*”—a question that essentially probes MSA ability from a subjective perspective. This apparent dissociation—between objectively superior MSA performance under fast DRC and higher subjective clarity ratings under slow DRC—initially seems contradictory. This discrepancy may be explained by listeners’ perceptual goals during holistic passive listening: potentially, attention naturally gravitates towards the more salient components, that is, those components of the mixture at higher sound levels (i.e., positive TMRs). Accordingly, as listeners were not explicitly instructed to focus on low-level instruments, it is likely that self-directed attention during the quality assessment was limited to more salient elements—those already advantaged under slow DRC. In these conditions, slow DRC then not only facilitates their extraction but also introduces fewer artefacts and distortions, while maintaining the temporal envelope of the signal. Paradoxically, fast DRC may have enabled audibility of less salient, low-level instruments, but the resulting increase in perceptual complexity and potential distortion may have led to lower subjective ratings. In a sense, listeners cannot judge what they cannot hear, and thus may have rated slow DRC higher based on the clarity of the dominant elements alone.

While speculative, this interpretation bears important implications for hearing aid fitting practices. These observations suggest that hearing aid optimization must account not only for objective performance metrics, but also for perceptual goals and subjective listening preferences. Specifically, fitting strategies that prioritize naturalness—such as slower DRC—may be better suited for holistic, enjoyment-oriented listening, whereas performance-oriented contexts, such as analytical music listening or scene parsing, may benefit from faster DRC settings that maximize the perceptual accessibility of softer or masked elements, thereby allowing access to all structural layers of the music, even at the expense of auditory artefacts and distortion introduced by fast DRC. Future research may extend to disentangle the influence of perceptual salience and listener intent on subjective versus objective measures of MSA by systematically varying the acoustic prominence of musical components to probe attentional allocation, or by contrasting active versus passive listening conditions to better characterize individual listening strategies and preferences. Such approaches may deepen our understanding of how perceptual priorities interact with scene-specific dynamics and DRC preferences, and, in turn, inform more personalized and context-sensitive hearing aid configurations.

### Listener-Specific Effects of Compression Speed Benefit

Individual variabilities in amplification benefits derived from compression settings within the same task further complement the present account. That is, the most beneficial DRC settings do not only depend on the TMR of the stimuli, but also on individual differences. A strong positive relationship was found between the benefit scores in slow and fast DRC conditions for the sound quality ratings, with notable consistency across participants. The same could be observed for the speech-in-noise task. This suggests that, for these tasks, individuals who benefit from one DRC setting also tend to benefit from the other, albeit to differing magnitudes. In contrast, however, the relationship for MSA was weak and nonsignificant: while fast DRC generally yielded better results on average, many listeners exhibited pronounced benefits with one DRC setting but little to no benefit with the other. Some participants even showed no improvements in MSA performance with amplification under either DRC setting. Accordingly, even within the same individual in the same task, improvements from amplification in one DRC setting do not necessarily translate to improvements in another DRC setting.

However, individual differences are not restricted to variability in responses to DRC effects within the same task, but are also observed across tasks. While the mixed-effects analysis predicting performance in one task from the outcomes of the others indicated that individuals who perform well in one domain tend to also perform well in others, substantial differences emerged in how much participants benefited from either DRC setting across tasks. That is, examination of individual-level outcomes revealed that the amplification benefit in one task does not necessarily correlate with the benefit to another, indicating considerable variability in how participants benefited from amplification across tasks. Specifically, only four participants showed alignment in their most beneficial DRC setting across tasks, consistently benefiting most from the slow DRC configuration. Most participants, however, achieved their best results with different DRC settings across tasks, reflecting the task-specific nature of the most beneficial compression setting based on cognitive and perceptual requirements. Notably, the highest agreement was observed between sound quality ratings and speech-in-noise tasks, with 18 participants showing the greatest benefit in the slow DRC condition for both tasks. This finding aligns with the results of the correlational analysis, where only the slow DRC condition showed a (marginal) relationship between SQR and SRT benefits.

Together, these findings highlight both the importance of individual variability and task-specificity in understanding DRC effects. That is they underscore that DRC influences on listeners’ ASA abilities are not only highly task-dependent but also vary significantly across individuals, even within the same task (see also Lunner et al., 1997; Moore et al., 2011; Ricketts et al., 2008). The potential of individualized hearing aid settings is further demonstrated by a comparison of MSA performance between hearing aid users and a reference population from the calibration study (Hake et al., 2023). In the unaided condition, hearing aid users performed at the lower end of the distribution for MSA, with a mean score of −.68, placing them below 75.2% in performance in comparison to the reference population. However, when equipped with their respective most beneficial DRC settings, their performance improved substantially, reaching a mean score of .003. This improvement elevated them to a level where only 49.9% of the reference population scored better, indicating that tailored hearing aid settings can restore MSA abilities to near-“normal” levels for many users. Nonetheless, the variability in benefit remains substantial, with some users experiencing no significant improvement or even showing better performance in the unaided condition. This heterogeneity emphasizes the critical role of personalization in hearing aid design for enhancing music perception, pointing to the need for deeper investigation into the individual factors that drive these differences.

Indeed, individual differences have been shown to affect compression settings outcomes. For example, despite the well-established dependency of the compression ratio and gain as a function of hearing loss (Scollie et al., 2005), previous research has shown that individuals with higher cognitive abilities are better able to benefit from fast compression, likely due to their ability to take advantage of temporal dips in the background sound (Gatehouse et al., 2006; Moore, 2008). In this study, we observed that participants with higher hearing thresholds (i.e., more severe hearing loss) experienced greater benefits from hearing aids across all tasks. Additionally, previous experience with hearing aids (that is longer history of hearing aid usage) was associated with improved performance, likely reflecting adaptation processes over time, a finding widely supported in the literature. Previous research has shown that long-term adaptation to DRC compression improves not only speech intelligibility but also performance in music-related tasks, such as melody identification (e.g., Uys & Latzel, 2015; Uys et al., 2016). However, it remained unclear whether these benefits extend to unfamiliar, ecologically valid musical mixtures—a question addressed in the present study. An important extension of the present findings would be to contrast the benefits of DRC processing with listening to uncompressed, live music. Future research should also consider item-level analyses to examine the effects of dynamic range and its interaction with both speech and music (across behavioral performance and subjective quality) under different hearing aid settings. This approach would allow for a more nuanced understanding of how dynamic range variability in the signal modulates perceptual outcomes across conditions.

Importantly, our results showed no effect of age, gender, or musical training on total amplification benefit (see also Knudsen et al., 2010). Additional factors, such as hearing aid usage history (including DRC type) and subclinical auditory processing deficits, such as hidden hearing loss, may also contribute to variability in ASA performance. Future research should explore these individual factors in more detail, including assessments of cognitive abilities, auditory processing skills, and detailed hearing aid experiences. Such investigations could clarify the variability in MSA benefit and inform personalized hearing aid fitting strategies that balance sound quality and selective listening needs across different auditory environments.

### Limitations

Several limitations of this study should be acknowledged. First, the participants’ degree of familiarity with different DRC settings was not assessed. Differences in, or lack of, acclimatization to the various DRC modes could have affected performance, as familiarity with certain compression speeds might impact auditory perception. Future studies should incorporate prior exposure to each DRC setting to control for potential adaptation effects. Second, the playback level used during testing was fixed and not varied across conditions. As compression is inherently level-dependent, the use of a single playback level may not capture the full range of effects that DRC settings have on auditory perception. Future studies should include multiple playback levels to better understand how DRC settings interact with level to influence performance and subjective experiences. Third, audibility was explicitly addressed during task-specific training sessions during which participants confirmed reliable perception of target signals and an acoustical analysis of the stimuli (see Figure A11 in Supp. Mat.) provides additional evidence that critical acoustical components were presented above individual hearing thresholds. It yet remains possible that limitations in audibility contributed to individual differences in performance. Future research should address this issue, for example, by including an additional amplified condition without dynamic range compression, thereby providing a baseline for disentangling the effects of basic audibility from those of compression processing.

Additionally, this study's operationalization of musical sound quality was limited. While recent research has identified seven key perceptual attributes of music audio quality for hearing aid users—clarity, harshness, distortion, spaciousness, treble strength, middle strength, and bass strength (Bannister et al., 2024)—we assessed clarity, sharpness (corresponding to “harshness”), and fullness, along with an overall quality rating. Incorporating a standardized set of perceptual attributes in future research, such as those proposed by the Bannister et al., (2024), could enhance the comparability and interpretability of findings across studies and provide a more comprehensive understanding of how DRC settings influence music perception. Using these attributes in subjective questionnaires could also complement prior exposure to DRC modes, offering a deeper insight into listeners’ perceptual experiences in music listening. Importantly, the present study's rating procedure may have introduced a degree of common method bias. All sound quality attributes were assessed immediately after each stimulus using similar Likert-scale formats and identical response timing and structure, which may have inflated correlations between ratings of different perceptual attributes. Consequently, the observed associations between dimensions such as sharpness and overall quality may partly reflect procedural artefacts rather than true perceptual relationships. Future research should seek to minimize this bias by varying the response modalities, temporally separating ratings of different attributes, randomizing attribute order across trials, or using alternative evaluation methods such as forced-choice paradigms to more robustly capture distinct perceptual dimensions.

Finally, while excerpts from the same source database were used for both the MSA and SQR tasks, the specific tracks differed across tasks. This design choice precluded direct item-level comparisons between subjective quality ratings and scene analysis performance. Thus, some differences between tasks may partly reflect stimulus-specific variation that was not systematically controlled for.

## Conclusion

Our results indicate that a one-size-fits-all approach to hearing aid dynamic range compression settings may not be optimal for music processing. Instead, different auditory tasks may benefit from different compression strategies, and hearing aid settings should be optimized depending on the specific needs and benefits of the listener. Additionally, the observed variability in amplification benefits for music stimuli across participants may reflect individual differences in auditory processing or cognitive abilities, underscoring the importance of personalized hearing aid fitting and tuning. Understanding the interaction between individual differences and hearing aid settings will be critical for optimizing auditory outcomes for hearing aid users, particularly in rich musical scenes.

## Supplemental Material

sj-docx-1-tia-10.1177_23312165251368669 - Supplemental material for Perception of Recorded Music With Hearing Aids: Compression Differentially Affects Musical Scene Analysis and Musical Sound Quality Supplemental material, sj-docx-1-tia-10.1177_23312165251368669 for Perception of Recorded Music With Hearing Aids: Compression Differentially Affects Musical Scene Analysis and Musical Sound Quality by Robin Hake, Michel Bürgel, Christophe Lesimple, Matthias Vormann, Kirsten C. Wagener, Volker Kuehnel and Kai Siedenburg in Trends in Hearing
