# Supplementary material for: Perception of Recorded Music With Hearing Aids: Compression Differentially Affects Musical Scene Analysis and Musical Sound Quality
Source: Trends Hear. 2025 Aug 25;29:23312165251368669. doi: 10.1177/23312165251368669 (PMC12378302; doi:10.1177/23312165251368669)
Supplement: sj-docx-1-tia-10.1177_23312165251368669 - Supplemental material for Perception of Recorded Music With Hearing Aids: Compression Differentially Affects Musical Scene Analysis and Musical Sound Quality [file sj-docx-1-tia-10.1177_23312165251368669.docx]

**Supplementary Material**

**Tables**

**Table A1**

*Sound excerpts used within the sound quality rating task*

| **Genre** | **Composer** | **Piece** | **Source** |
| --- | --- | --- | --- |
| Classic | Ludwig van Beethoven | Overture “Coriolan”, Op. 62 | https://www.youtube.com/ watch?v=DzINFjNPmqI |
|  | Joseph Haydn | Symphony No. 31 in D major Hob. I 31 – “Hornsignal”:  I. Allegro | https://www.youtube.com/ watch?v=kcVZQel5NqQ |
|  | Felix Mendelssohn | Symphony n3 in A minor, Op.56 | https://www.youtube.com/ watch?v=RnkYSNA0-tA |
|  | Wolfgang Amadeus Mozart | Fagottkonzert B-Dur K191 Allegro | https://www.youtube.com/ watch?v=kQgLCsbaDk8 |
|  | Antonio Salieri | Sinfonia in D Major, 'Veneziana' -  1. Allegro assai | https://www.youtube.com/ watch?v=Cayi95DvRNI |
| Pop | Dead Milkmen | Prisoners Cinema | Medley DB |
|  | Hop Along | Sister Cities | Medley DB |
|  | Invisible Familiars | Disturbing Wildlife | Medley DB |
|  | Patrick Talbot | Fool | Medley DB |
|  | Strand of Oaks | Spacestation | Medley DB |
|  |  |  |  |

*Note.* Online videos (youtube.com) last accessed on: 29.10.2024.

**Table A2**

*Sound quality dimensions of the sound quality rating task in the original language (German)*

| **Dimension** | **Prompt** | **Lowest rating** | **Highest rating** |
| --- | --- | --- | --- |
| Lautheit | Wie laut war der Klang? | Leise | Laut |
| Schärfe | Wie scharf/schrill oder weich war der Klang? | Weich | Scharf |
| Sattheit/Völle | Wie voll war der Klang? | Dünn | Voll |
| Heraushörbarkeit | Wie deutlich konnten Sie die einzelnen Instrumente voneinander unterscheiden? | Undeutlich | Sehr klar |
| Gesamtqualität | Wie gut war die allgemeine Klangqualität der Musik? | Schlecht | Gut |

*Note.*

**Table A3**

*Descriptive Statistics and Correlations for Study Variables*

| Model | Variable | AIC | BIC | R2 (cond.) | R2 (marg.) | ICC | Sigma |
| --- | --- | --- | --- | --- | --- | --- | --- |
|  | **Musical scene analysis** |  |  |  |  |  |  |
| A1 | MSA ~ condition + (1 \| ID) | 192.8 | 205.8 | .322 | .1 | .247 | .552 |
| A2^a^ | MSA ~ condition + order + (1 \| ID) | 193.7 | 209.2 | .327 | .11 | .248 | .551 |
| A3^b^ | MSA ~ condition * order + (1 \| ID) | 197.2 | 217.9 | .341 | .11 | .261 | .552 |
| A4^c^ | MSA ~ condition + tinnitus  + (1 \| ID) | 196.5 | 209.9 | .331 | .1 | .259 | .552 |
| A5 ^d^ | MSA ~ condition + musical training + (1 \| ID) | 201.9 | 214.4 | .331 | .1 | .259 | .552 |
|  | **Sound quality ratings models** |  |  |  |  |  |  |
| B1 | SQR ~ condition  + (1 \| ID) + (1 \| stim) | 3779 | 3808 | .357 | .09 | .294 | 1.53 |
| B2^e^ | SQR ~ condition + genre  + (1 \| ID) + (1 \| stim) | 3778 | 3779 | .358 | .1 | .289 | 1.53 |
| B3^f^ | SQR ~ condition * genre  + (1 \| ID) + (1 \| stim) | 3781 | 3825 | .36 | .1 | .289 | 1.53 |
| B4^g^ | SQR ~ condition + genre  + (1 \| ID) + (1 + condition \| stim) | 3787 | 3787 | *NA* | .13 | *NA* | 1.53 |
| B5^h^ | SQR ~ condition + tinnitus  + (1 \| ID) + (1 \| stim) | 3778 | 3778 | .362 | .12 | .277 | 1.53 |
| B6^i^ | SQR ~ condition + musical training  + (1 \| ID) + (1 \| stim) | 3782 | 3821 | .364 | .1 | .295 | 1.53 |
|  | **Speech reception thresholds** |  |  |  |  |  |  |
| C1 | SRT ~ condition + (1 \| ID) | 383.7 | 386.6 | .755 | .135 | .717 | 1.12 |
| C2^j^ | SRT ~ condition + tinnitus + (1 \| ID) | 384.6 | 400.1 | .76 | .132 | .724 | 1.12 |
| C3^k^ | SRT ~ condition + training + (1 \| ID) | 385.7 | 401.3 | .76 | .137 | .722 | 1.12 |

*Note.* Total *N* = 99. CI = confidence interval; *LL* = lower limit; *UL* = upper limit. Compared to the simpler model, the model fit did not significantly improve the model fit (^a^χ^2^ = 1.12, p = .29; ^b^χ^2^ = .52, p = .77, ^c^χ^2^ = .003, p = .96*,* ^d^χ^2^ = .003, p = .87*,* ^e^χ^2^ = 7.85, p = .005, ^f^χ^2^ = .75, p = .69, ^g^χ^2^ = 1.48, p = .22, ^h^χ^2^ = 3.02, p = .22, ^i^χ^2^ = .003, p = .87*,* ^j^χ^2^ = .003, p = .96, ^d^χ^2^ = .003, p = .61). ^f^Boundary issue or singular fit, meaning the model may be overfitted or too complex.

**Table A4**

*Final model (A1) summary for the musical scene analysis (MSA) ability scores*

| Effect | Estimate | *SE* | 95% CI | | *p* |
| --- | --- | --- | --- | --- | --- |
|  |  |  | *LL* | *UL* |  |
| **Model A1:** **MSA ~ condition + (1 \| subject)** |  |  |  |  |  |
| Intercept | -.68 | .11 | -.9 | -.46 | < .001 |
| Slow DRC | .19 | .14 | -.08 | .46 | .175 |
| Fast DRC | .51 | .14 | .25 | .78 | < .001 |
| Random effects |  |  |  |  |  |
| Subject (Intercept) | .1 | .32 (SD) | |  |  |
| Residual | .3 | .55 (SD) | |  |  |

*Note.* Total Observations = 99. CI = confidence interval; *LL* = lower limit; *UL* = upper limit.

**Table A5**

*The correlation matrix for the test battery*

| Variable | Overall quality | Clearness | Fullness | Loudness | Sharpness |
| --- | --- | --- | --- | --- | --- |
| Overall quality | - | .69^***^ | .59^***^ | .44^***^ | .20^***^ |
| Clearness |  | - | .53^***^ | .48^***^ | .26^***^ |
| Fullness |  |  | - | .43^***^ | .10^**^ |
| Loudness |  |  |  | - | .65^***^ |
| Sharpness |  |  |  |  | - |

**Note**. Above the diagonal are the Pearson correlations calculated from a
complete pair of values, which are significant at ^*^p < .05, ^**^p < .01,
 ^***^p < .001. N = 988.

**Table A6**

*Final model (B2) summary for the sound quality rating (SQR) factor scores*

| Effect | Estimate | *SE* | 95% CI | | *p* |
| --- | --- | --- | --- | --- | --- |
|  |  |  | *LL* | *UL* |  |
| **Model B2:** **SQR ~ condition + genre + (1 \| subject) + (1 \| stimuli)** | | |  |  |  |
| Intercept (Unaided) | 5.76 | .2 | 5.36 | 6.16 | .37 |
| Slow DRC | 1.4 | .12 | 1.17 | 1.64 | <.001 |
| Fast DRC | .62 | .12 | .38 | .85 | <.001 |
| Genre: Pop | -.33 | .15 | -.62 | -.05 | .023 |
| Random effects |  |  |  |  |  |
| Subject (Intercept) | .87 | .93 (SD) | |  |  |
| Stimuli (Intercept) | .09 | .29 (SD) | |  |  |
| Residual | 2.35 | 1.53 (SD) | |  |  |

*Note.* Total Observations = 990. CI = confidence interval; *LL* = lower limit; *UL* = upper limit.

**Table A7**

*Final model (C1) summary for the speech reception thresholds (SRT) from the Göttinger Sentence Test*

| Effect | Estimate | *SE* | 95% CI | | *p* |
| --- | --- | --- | --- | --- | --- |
|  |  |  | *LL* | *UL* |  |
| **Model B2:** **SRT ~ condition + (1 \| subject)** | | |  |  |  |
| Intercept (Unaided) | .49 | .36 | -.23 | 1.22 | .18 |
| Slow DRC | -1.94 | .28 | -2.49 | -1.4 | <.001 |
| Fast DRC | -.48 | .28 | -1.03 | 0.07 | .08 |
| Random effects |  |  |  |  |  |
| Subject (Intercept) | 3.18 | 1.8 (SD) | |  |  |
| Residual | 1.3 | 1.12 (SD) | |  |  |

*Note.* Total Observations = 990. CI = confidence interval; *LL* = lower limit; *UL* = upper limit.

**Table A8**

*Mixed effects model predicting MSA scores with SQR and SRT*

|  | **LSM estimate of MSA** | | | |
| --- | --- | --- | --- | --- |
| *Predictors* | *Estimates* | *std. Error* | *Statistic* | *p* |
| Intercept | -0.79 | 0.44 | -1.80 | 0.075 |
| SQR | 0.03 | 0.06 | 0.56 | 0.579 |
| SRT | -0.14 | 0.04 | -3.25 | **0.002** |
| **Random Effects** | | | | |
| σ^2^ | 0.56 | | | |
| τ_00_ _p_id_ | 0.05 | | | |
| τ_00_ _condition_ | 0.11 | | | |
| ICC | 0.23 | | | |
| N _p_id_ | 33 | | | |
| N _condition_ | 3 | | | |
| *Note*. Observations | 99 | | | |
| Marginal R^2^ / Conditional R^2^ | 0.132 / 0.331 | | | |

**Table A9**

*Mixed effects model predicting SQR scores with SRT and MSA*

|  | **LSM estimate of SQR** | | | |
| --- | --- | --- | --- | --- |
| *Predictors* | *Estimates* | *std. Error* | *Statistic* | *p* |
| Intercept | 6.26 | 0.32 | 19.84 | **<0.001** |
| SRT | -0.22 | 0.07 | -3.00 | **0.003** |
| MSA | 0.11 | 0.16 | 0.66 | 0.508 |
| **Random Effects** | | | | |
| σ^2^ | 1.33 | | | |
| τ_00_ _p_id_ | 0.36 | | | |
| τ_00_ _condition_ | 0.20 | | | |
| ICC | 0.29 | | | |
| N _p_id_ | 33 | | | |
| N _condition_ | 3 | | | |
| *Note*. Observations | 99 | | | |
| Marginal R^2^ / Conditional R^2^ | 0.129 / 0.385 | | | |

**Table A10**

*Mixed effects model predicting SRT scores with SQR and MSA*

|  | **LSM estimate of SRT** | | | |
| --- | --- | --- | --- | --- |
| *Predictors* | *Estimates* | *std. Error* | *Statistic* | *p* |
| Intercept | 0.95 | 0.91 | 1.04 | 0.301 |
| SQR | -0.24 | 0.11 | -2.15 | **0.034** |
| MSA | -0.42 | 0.17 | -2.47 | **0.016** |
| **Random Effects** | | | | |
| σ^2^ | 1.20 | | | |
| τ_00_ _p_id_ | 2.54 | | | |
| τ_00_ _condition_ | 0.71 | | | |
| ICC | 0.73 | | | |
| N _p_id_ | 33 | | | |
| N _condition_ | 3 | | | |
| *Note.* Observations | 99 | | | |
| Marginal R^2^ / Conditional R^2^ | 0.063 / 0.747 | | | |

**Table A11**

*Sociodemographic characteristics of participants at baseline*

| Baseline characteristic | with Tinnitus | | without Tinnitus | | Effect Estimate (*β*) | *p* | |
| --- | --- | --- | --- | --- | --- | --- | --- |
|  | *M* | SD | *M* | SD |  | |  |
| MSA | -.45 | .58 | -.45 | .72 | .01 | | .96 |
| SRT | -.27 | 2.44 | -.38 | 3.02 | -.11 | | .87 |
| SQR | 6.09 | .72 | 6.66 | .87 | .65 | | .052 |
| SRT |  |  |  |  |  | |  |

*Note. N* = 33. In total, 13 out of 33 participants (39%) reported experiencing some

form of tinnitus lasting longer than three months. Of these, seven participants reported

permanent tinnitus, two experienced tinnitus it only in quiet environments, three under

stress, and one reported it occasional experiences of tinnitus. No sig. effect was found
(see also Table A3).

**Figures**

**Figure A1**

**
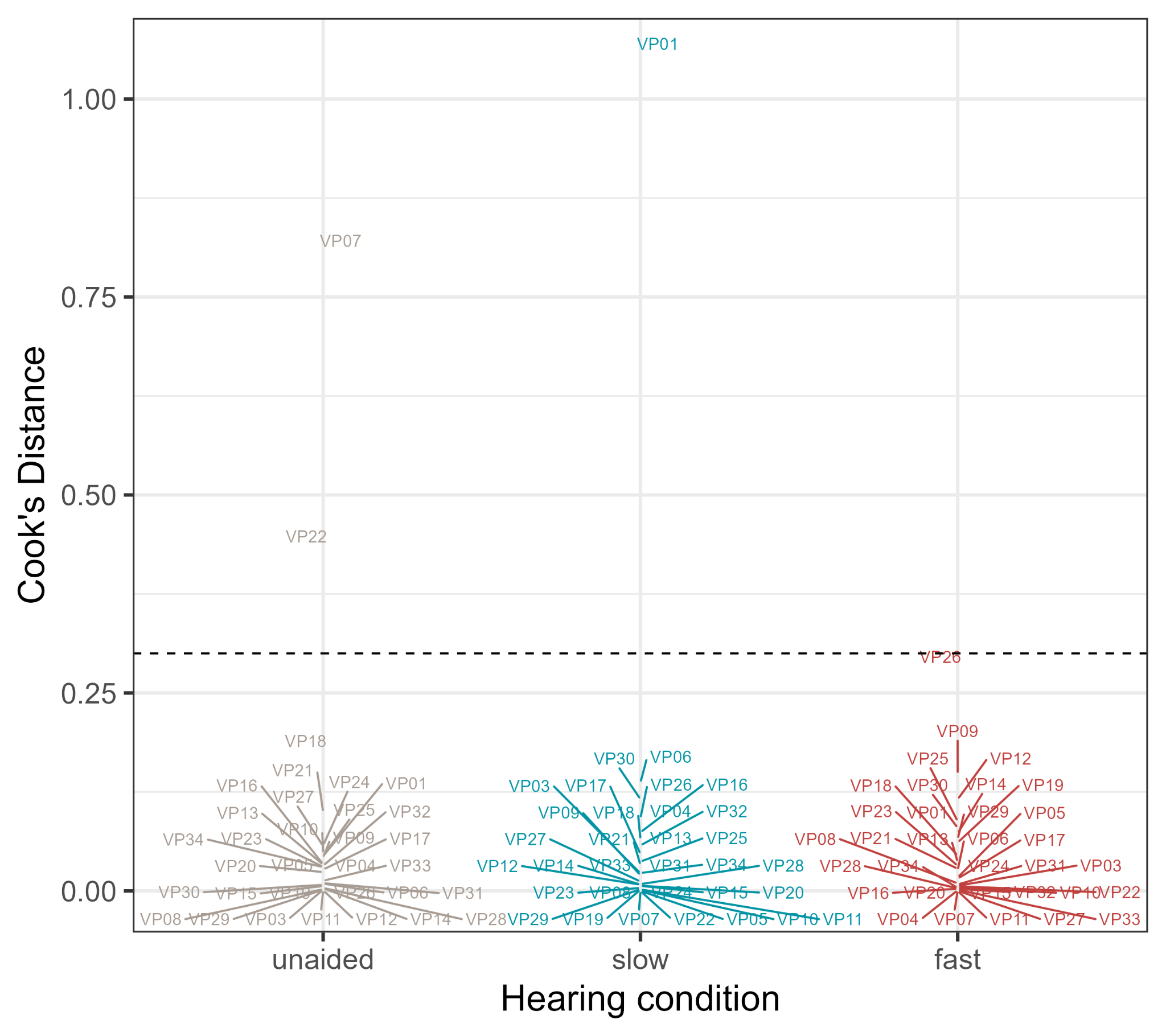
**

**Figure A1.** Cook's distance analysis for identifying influential data points. The dashed horizontal line indicates the threshold for exclusion, determined through visual inspection. Three data points exceeding this threshold (VP01 in the slow compression condition, VP07 and VP22 in the unaided condition) were subsequently excluded due to chance or near-chance performance on the primary task. These data points were replaced by imputation (see ‘Analysis’ section).

**Figure A2**


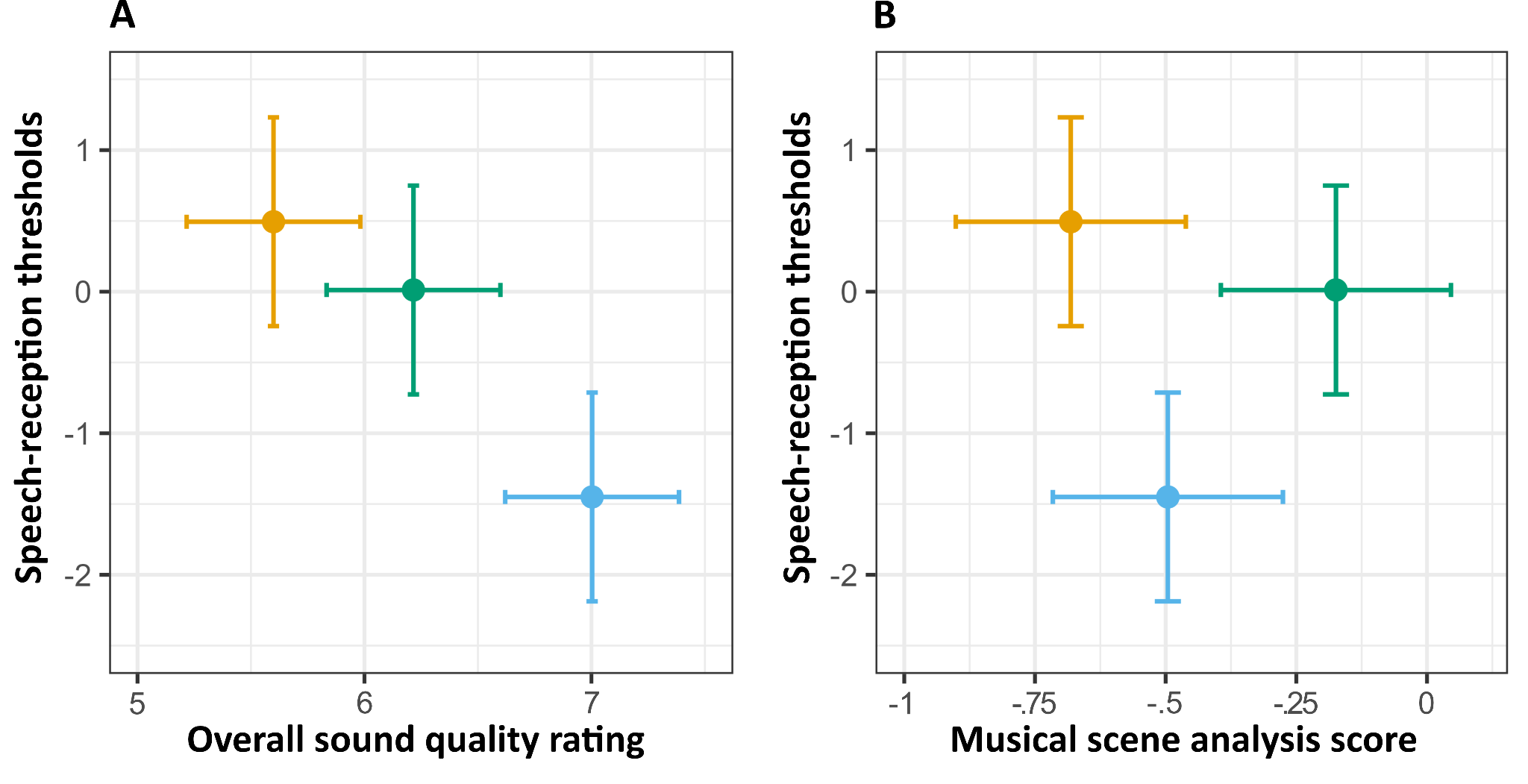
**Figure A2.** Estimated means from Model A1 for musical scene analysis (MSA) scores, Model B2 for sound quality rating factor (SQR) scores, and Model C1 for speech reception thresholds (SRT) across three conditions: slow (blue), unaided (yellow), fast (green). Panel A shows the relationship between SRT and SQR scores, while Panel B displays the relationship between SRT and MSA scores. Error bars represent 95% confidence intervals.

**Figure A3**


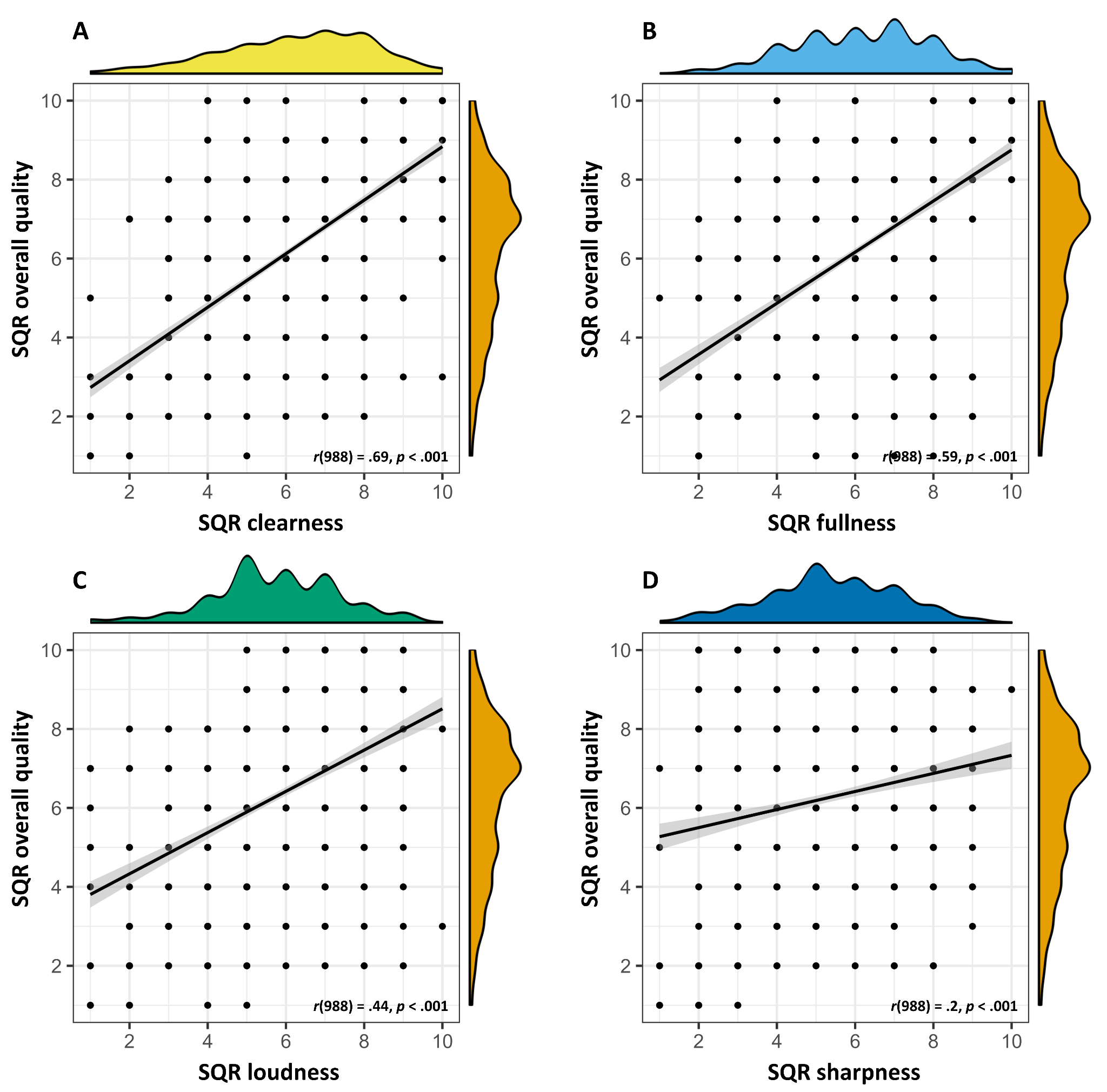


**Figure A3.** Relationship between overall sound quality ratings (SQR) and individual sound quality dimensions: (A) clearness, (B) fullness, (C) loudness, and (D) sharpness. Each plot displays a scatterplot with a linear regression line (solid black) and a 95% confidence interval (shaded area) for each dimension in relation to overall quality. Density plots for each dimension are displayed along the top and right margins to illustrate the distribution of ratings. Pearson correlation coefficients are shown within each panel, indicating significant positive correlations between overall quality and each dimension.

**Figure A4**


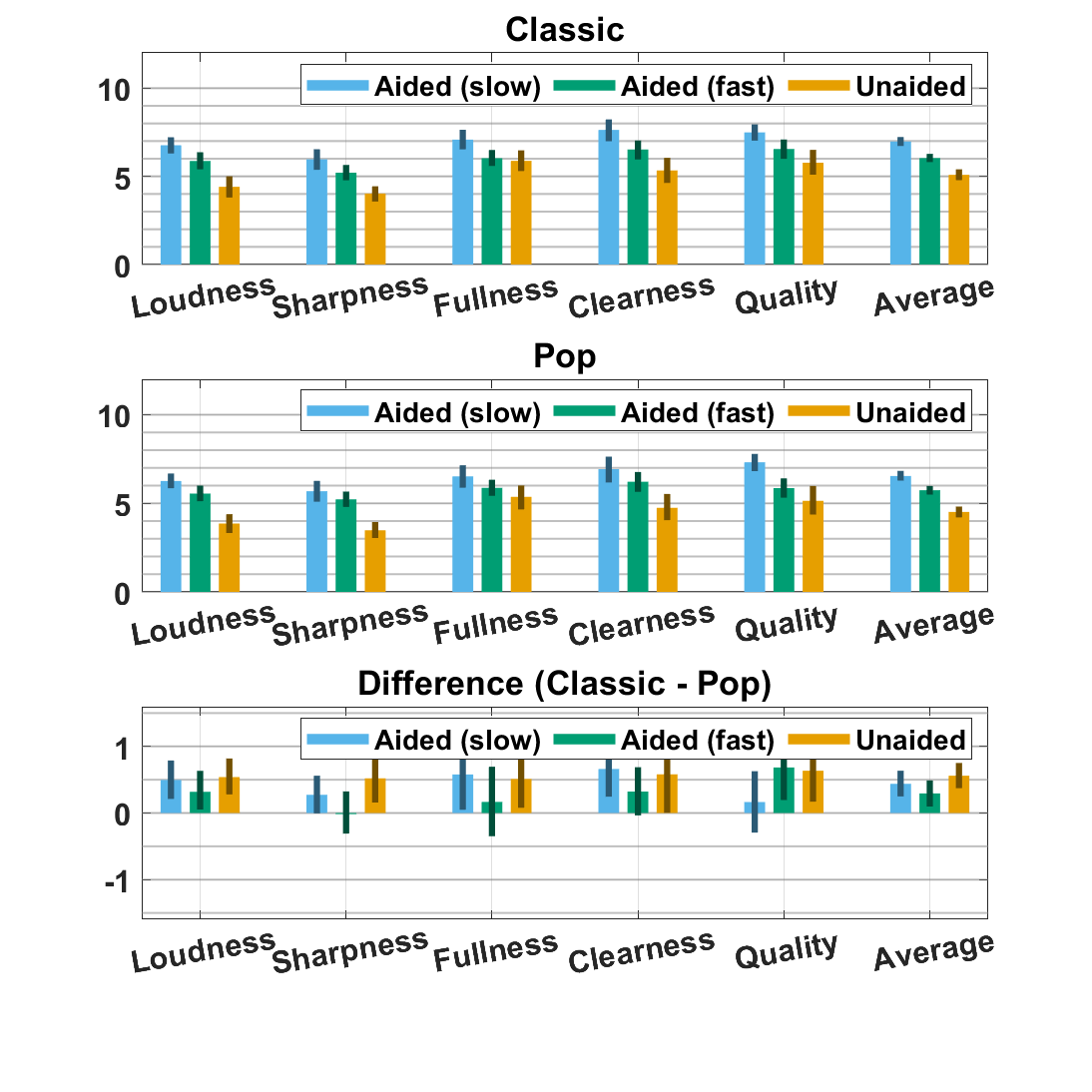


**Figure A4.** Comparative assessment of sound quality attributes under different listening conditions. The bar graphs display ratings for various sound quality attributes (Loudness, Sharpness, Fullness, Clearness, and Overall Quality) across two musical genres: Classical and Pop. The ratings were evaluated under three conditions: unaided, aided with slow compression (Aided slow), and aided with fast compression (Aided fast). The top two panels show the mean ratings for each attribute for Classical and Pop music, respectively. The bottom panel displays the mean differences in ratings between Classical and Pop music for each attribute and condition. Error bars represent standard deviations. Higher values indicate more positive evaluations of the sound quality attributes.

**Figure A5**


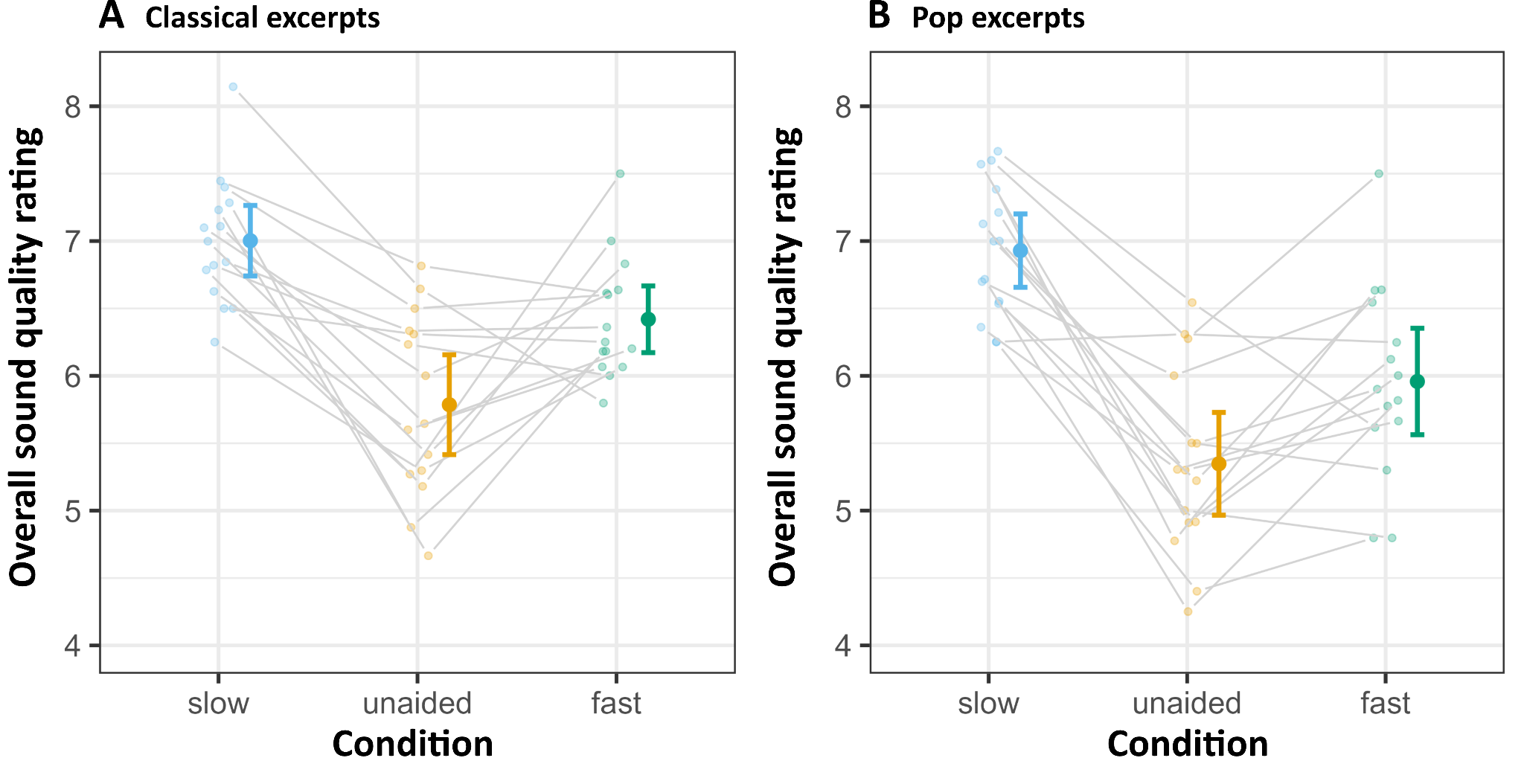
**Figure A5.** Mean overall sound quality rating (SQR) factor scores across three listening conditions (slow, unaided, and fast) for classical (Panel A) and pop excerpts (Panel B). Individual shaded dots represent SQR scores averaged over stimuli. Accordingly, average results of the same stimuli are displayed for the three listening conditions. Error bars represent 95% confidence intervals. Note significance at *p < .05, **p < .01, ***p < .001.

**Figure A6**


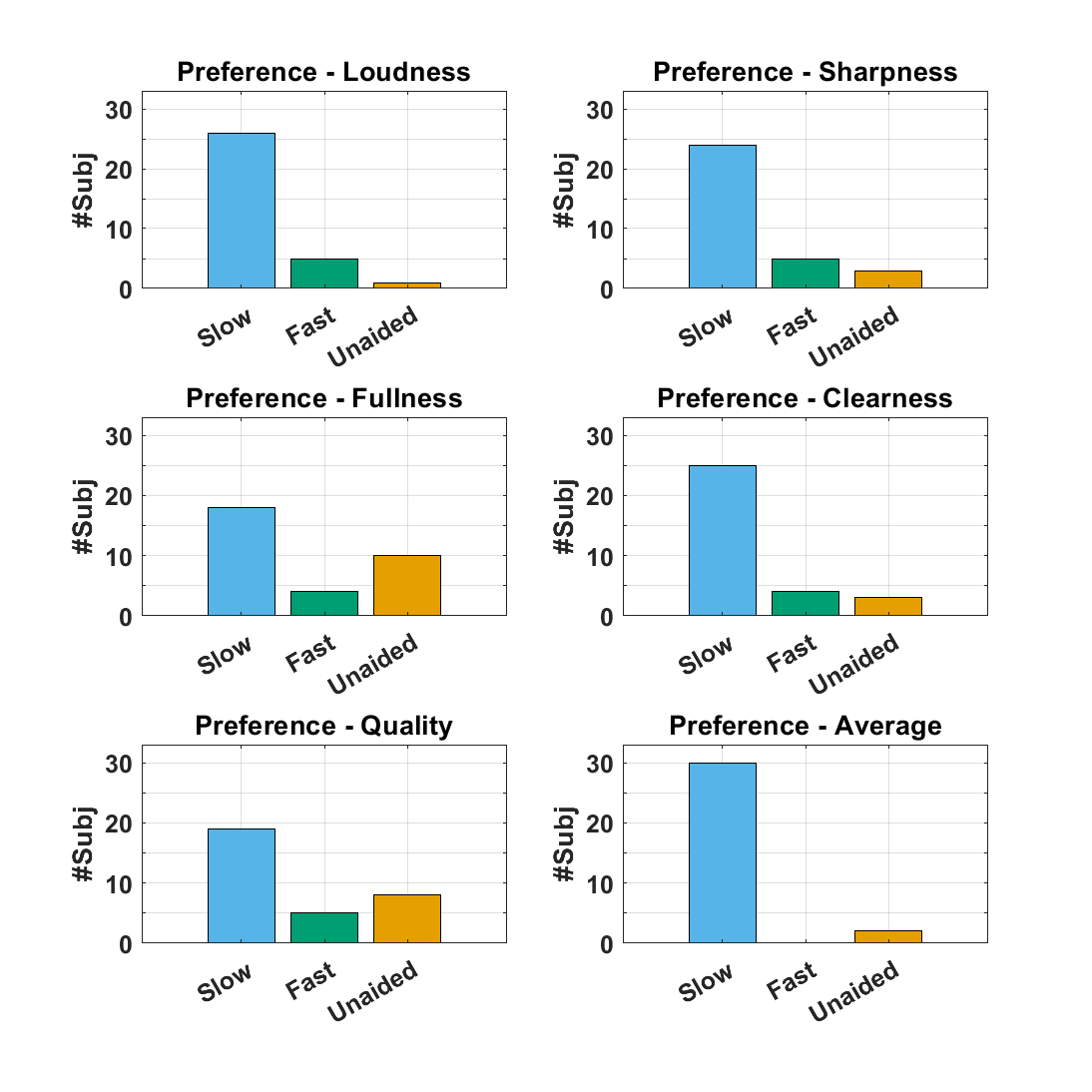


**Figure A6.** Preferred listening condition for each sound quality dimension. The bar graphs display the preferred condition across various sound quality dimensions (Loudness, Sharpness, Fullness, Clearness, and Overall Quality), averaged across two musical genres: Classical and Pop. Ratings were evaluated under three conditions: unaided, aided with slow compression (slow), and aided with fast compression (fast). Each panel represents a different sound quality dimension. The bars indicate the number of subjects who, on average, rated each condition as the highest. Higher values reflect a greater number of subjects preferring that condition. Please note that for loudness and sharpness, the bipolar scales used (e.g., 'very soft' to 'very loud') indicate extreme scores and may thus primarily reflect perceived intensity rather than an explicit preference.

**Figure A7**


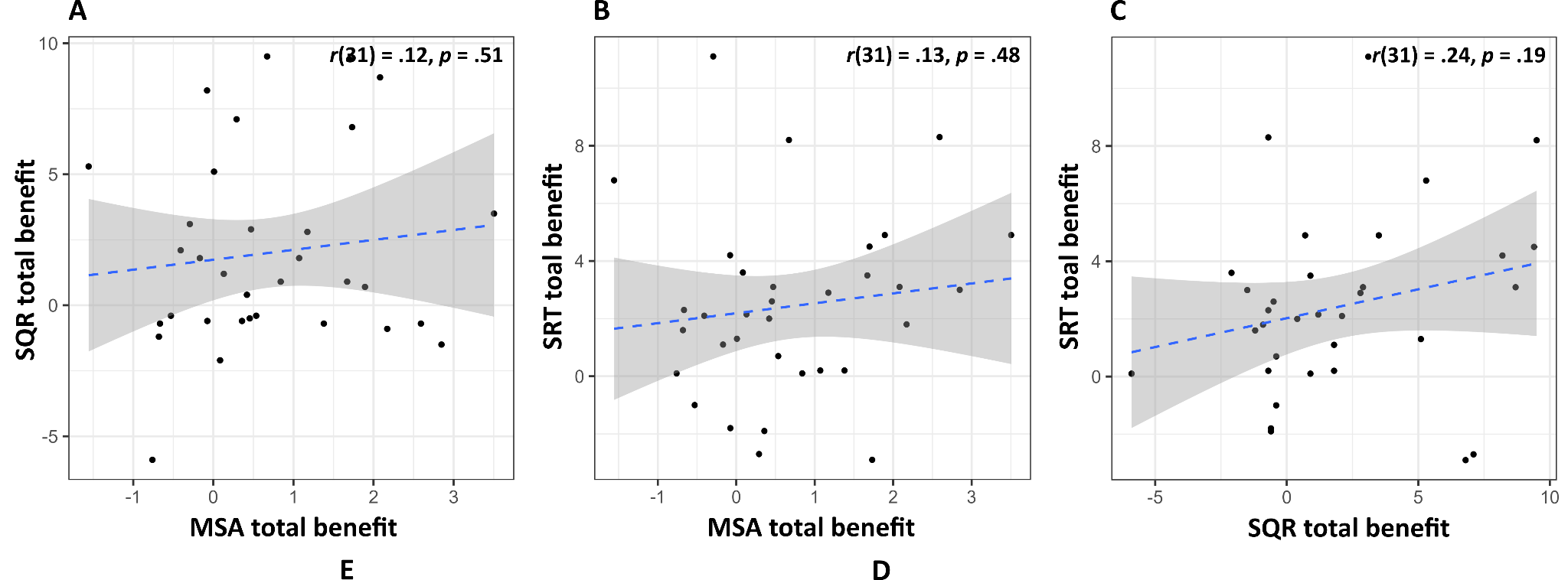
**Figure A7.** Scatterplots showing the relationships between total benefit scores across three measures: (A) SQR (overall Sound Quality Rating) and MSA (Musical Scene Analysis abilities), (B) SRT (Speech Reception Threshold in dB SNR) and MSA, and (C) SRT and SQR. Total benefit scores were calculated by subtracting the unaided condition scores from both the fast DRC and slow DRC condition scores, then summing these differences to create an aggregate do this analysis with the individuals best DRC in task 1 vs. best DRC setting in benefit measure. Each plot displays individual data points along with a linear regression line (dashed blue) and 95% confidence interval (shaded area). Pearson correlation coefficients and p-values are provided in each panel.

**Figure A8**

**
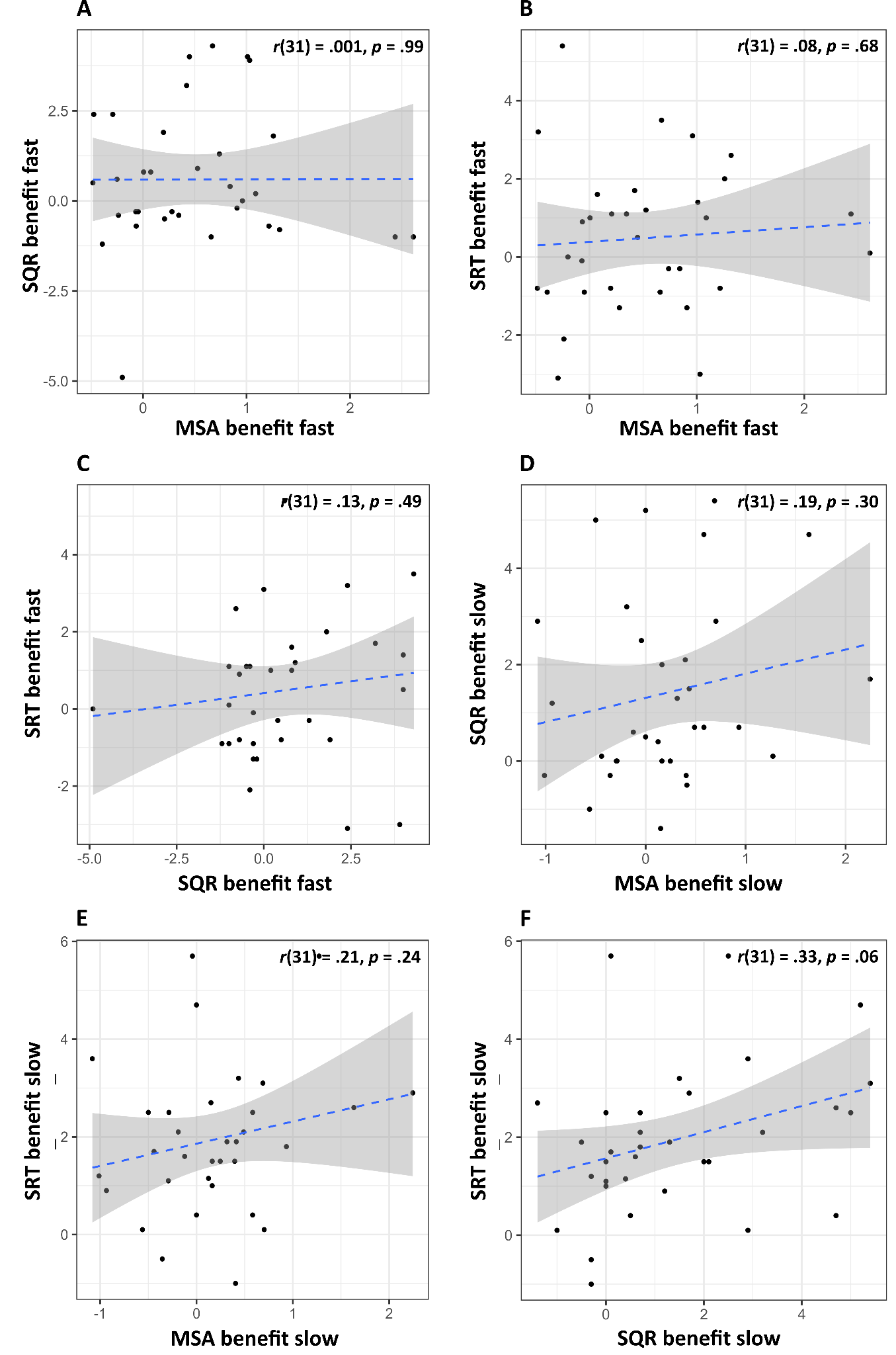

Figure A8.** Scatterplots illustrating the relationships between benefit scores across tasks and DRC settings (fast and slow). Panels (A), (B), and (C) depict the correlations between benefit scores under the fast DRC condition: (A) overall sound quality rating (SQR) and Musical scene analysis abilities (MSA), (B) Speech reception threshold (SRT) and MSA, and (C) SRT and SQR, Panels (D), (E), and (F) depict correlations for the slow DRC condition: (D) SQR and MSA, (E) SRT and MSA, and (F) SRT and SQR. Each plot includes a dashed blue regression line with a 95% confidence interval shaded in gray. Pearson correlation coefficients and p-values are provided in each panel.

**
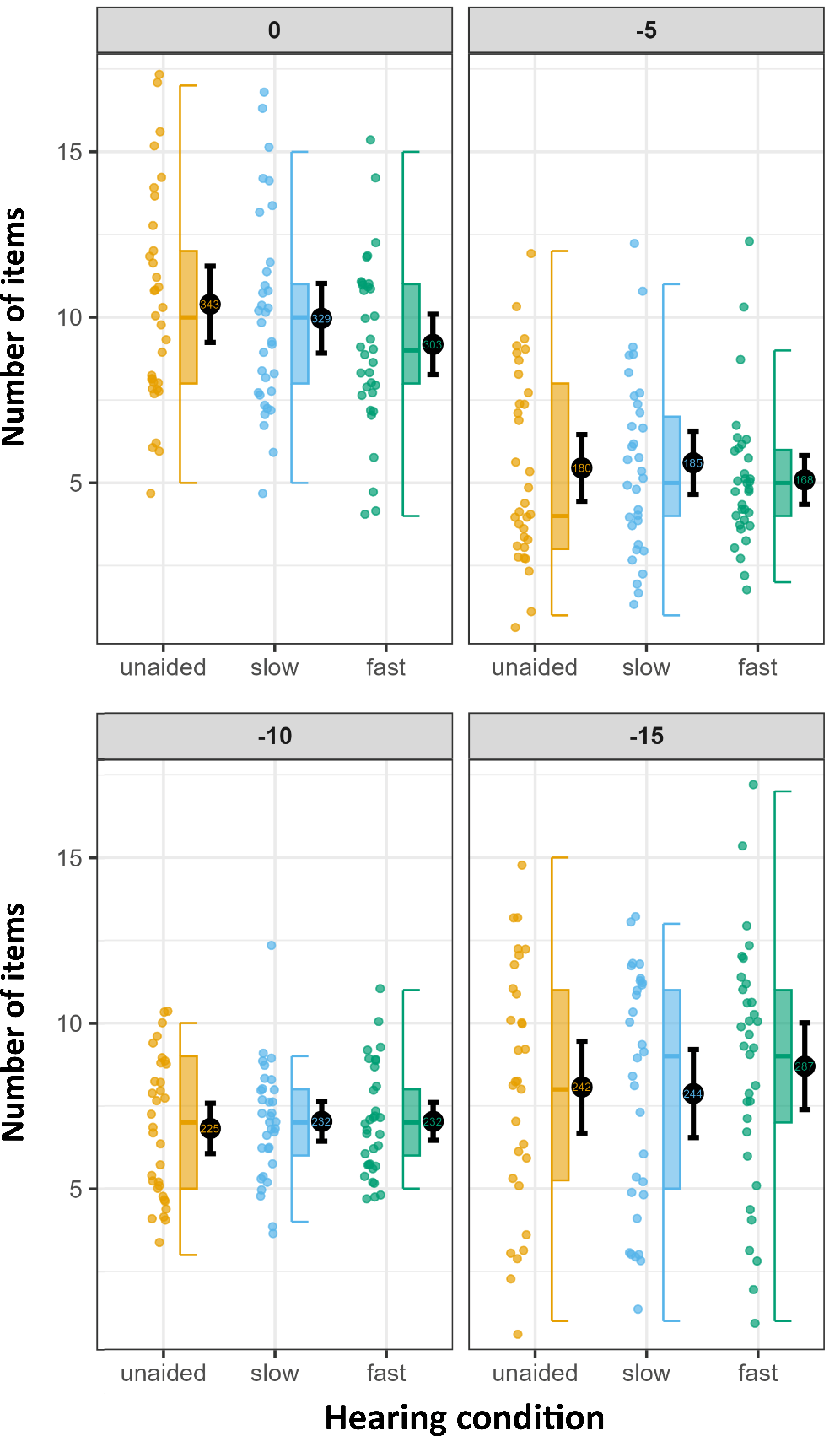

Figure A9.** Number of correctly identified items per participant by listening condition and TMR level. Each panel represents one TMR level (0, –5, –10, –15 dB). Within each panel, distributions are shown separately for the three listening conditions: unaided, slow DRC, and fast DRC. Individual participant scores are displayed as half-violin scatterplots (left side), while the corresponding distributions are shown as half boxplots (right side). Overlaid black point-ranges indicate group means with 95% confidence intervals, and accompanying numerals indicate the number of contributing participants per condition.


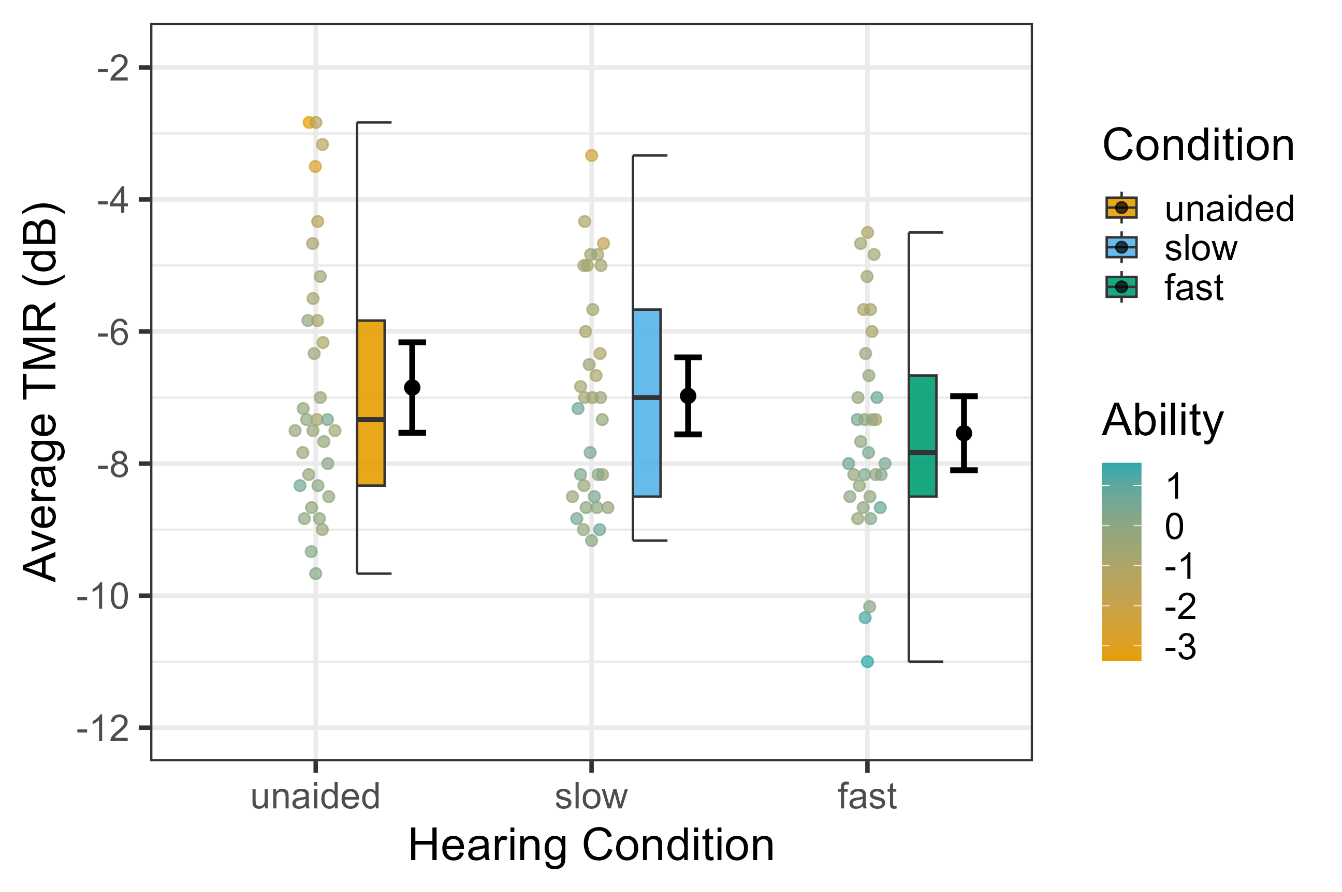
**Figure A10.** Distribution of average target-to-mixture ratios (TMRs) by hearing condition. Individual data points represent each participants mean TMR per condition and are colour-coded by MSA performance ability, with higher scores shown in blue and lower scores in yellow. Boxplots display individual participant quantiles in the unaided, slow DRC, and fast DRC conditions. Overlaid black point-ranges represent group means with 95% confidence intervals. More negative TMRs indicate greater masking of the target signal within the mixture. A linear mixed-effects model was fitted to examine differences in average TMR across listening conditions. No significant differences in average TMR were observed between the unaided, slow DRC, and fast DRC conditions. Specifically, pairwise comparisons using estimated marginal means revealed no significant differences in average TMR across listening conditions (all p-values > .29). Specifically, the difference between the unaided and slow DRC conditions was not significant (β = 0.13, SE = 0.41, t(64) = 0.31, p = .76), nor between unaided and fast DRC (β = 0.69, SE = 0.41, t(64) = 1.68, p = .30), nor between slow and fast DRC (β = 0.57, SE = 0.41, t(64) = 1.37, p = .35)

*
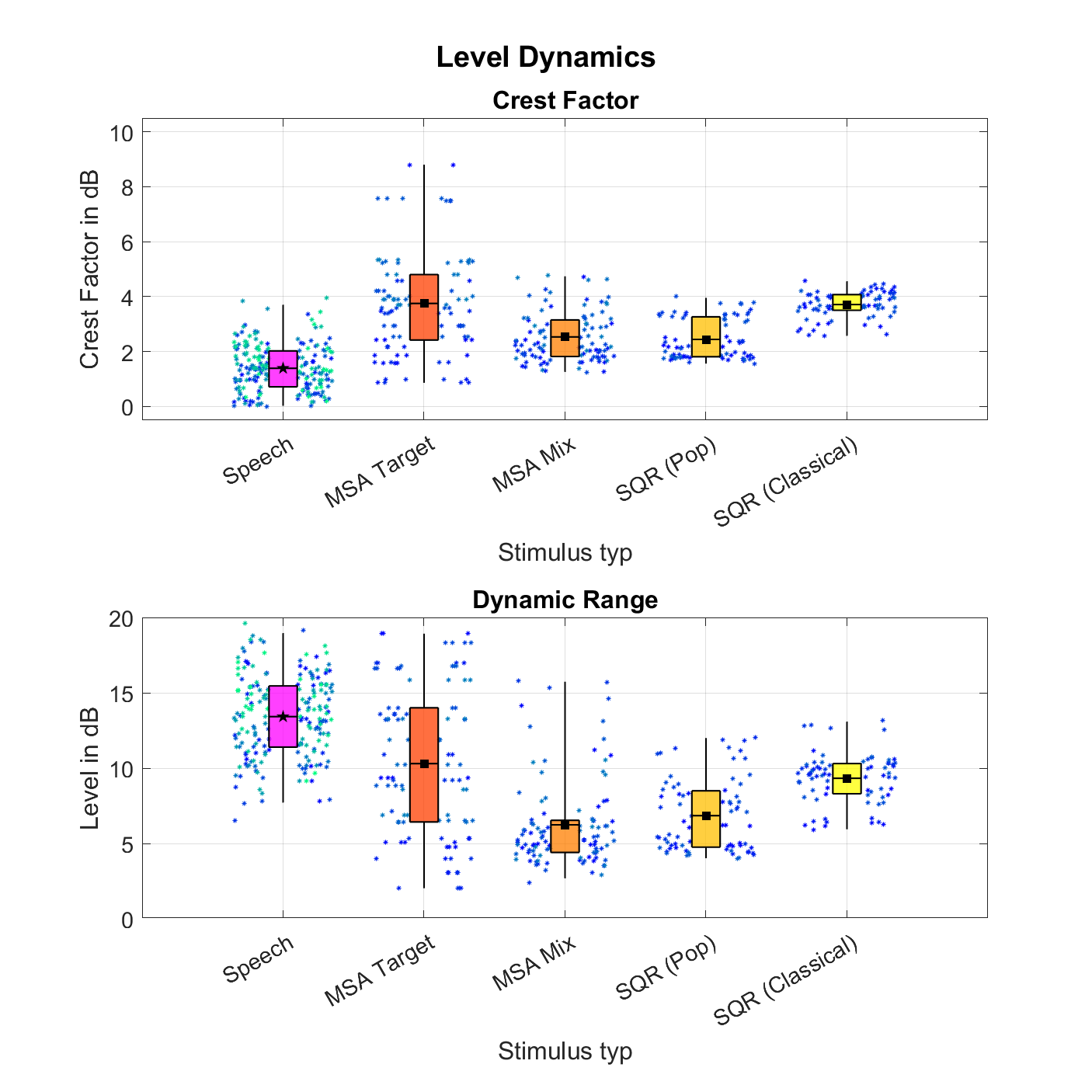
*

**Figure A11.** Sound level characteristics of the experimental stimuli, assessed via two dynamic-level metrics: crest factor, and dynamic range. The horizontal axis shows the five stimulus types: speech sounds from the GÖSA (SRT) corpus, excerpts of isolated target sounds and musical mixtures from the MSA task, and pop music as well as classical music excerpts from the SQR task. The vertical axis represents the respective values in dB. Each dot corresponds to an individual stimulus item. All stimuli were RMS-normalized to -20 dB FS prior to analysis. RMS levels were computed using a 100 ms moving window with 50 ms overlap. To avoid including extended silence at the beginning or end, only windows between the first and last time a level threshold was exceeded were considered. The threshold was set to
-40 dB relative to the maximum level. The dynamic range was evaluated as the level difference between the time window with the highest level and the lowest level. For the MSA stimuli, we focused on the final two seconds - corresponding to the target detection period - for both the isolated targets and the mixture signals. *Figure caption is continued on the next page.*

**Figure A11.** *(continued)* For the SQR stimuli, a bootstrap procedure with 100 iterations was applied to provide a balanced number of stimuli between the stimulus types. For the bootstrap, two-second excerpts were randomly sampled from the full 30-seconds to yield a metric comparable to the shorter MSA and speech stimuli. The isolated GÖSA (SRT) speech signals were only level-normalized, as they had a duration of approximately two seconds. It is important to note that this analysis diverges from the actual task presentation, where speech was embedded in noise with varying signal-to-noise ratios. Consequently, the effective dynamics experienced by participants during the task differ from those analyzed here. This was necessary because the dynamic range then depends on the individual, as the level of the noise is adjusted to the performance of the listener. Nevertheless, this approach offers insights into the dynamic properties of the clean speech signals and enables a first comparison with the music stimuli. The combined analysis of crest factor and dynamic range revealed distinct profiles across stimulus categories. Speech and isolated instruments exhibited a broad dynamic range, likely driven by silence and articulation pauses. Yet they differed in their crest factors, reflecting fewer transient peaks in speech. Music mixtures, both in the MSA task and the SQR pop excerpts, showed reduced dynamic variation and more compressed dynamic profiles, reflecting that they originated from the same source material. In contrast, classical music retained both a high crest factor and wide dynamic range. Speech signals exhibited the highest dynamic range but relatively low crest factors, indicating a pause-rich yet transient-poor structure. Participants benefited most from slow compression in the speech intelligibility task, likely because fast-acting compression amplifies low-level noise, thereby reducing the signal-to-noise ratio. Moreover, fast-acting compression tends to diminish intensity contrasts and reduce the modulation depth of speech, leading to distortion of temporal cues (see main article). In contrast, fast compression improved performance in the MSA task - potentially by enhancing the salience of transients and thus supporting auditory stream segregation. For the sound quality ratings, both pop and classical music benefited from slow DRC, despite their different dynamics. This suggests that perceptual preferences are less influenced by absolute dynamic characteristics, further underlining the importance of preserving naturalness and dynamic contrast.
